# Risk-Based Decision Making: A Systematic Scoping Review of Animal Models and a Pilot Study on the Effects of Sleep Deprivation in Rats

**DOI:** 10.3390/clockssleep3010003

**Published:** 2021-01-20

**Authors:** Cathalijn H.C. Leenaars, Stevie Van der Mierden, Ruud N.J.M.A. Joosten, Marnix A. Van der Weide, Mischa Schirris, Maurice Dematteis, Franck L.B. Meijboom, Matthijs G.P. Feenstra, André Bleich

**Affiliations:** 1 Institute for Laboratory Animal Science, Hannover Medical School, 30625 Hannover, Germany; VanderMierden.Stevie@mh-hannover.de (S.V.d.M.); Bleich.Andre@mh-hannover.de (A.B.); 2 Department for Health Evidence (Section HTA), SYRCLE, Radboud University Medical Centre, 6600 Nijmegen, The Netherlands; 3 Unit Animals in Science and Society, Population Health Sciences, Utrecht University, 3500 Utrecht, The Netherlands; F.L.B.Meijboom@uu.nl; 4 Netherlands Institute for Neuroscience, an Institute of the Royal Netherlands Academy of Arts and Sciences, 1000 Amsterdam, The Netherlands; R.Joosten@nin.knaw.nl (R.N.J.M.A.J.); marnixvanderweide@xs4all.nl (M.A.V.d.W.); mschirris@gmail.com (M.S.); m.feenstra@nin.knaw.nl (M.G.P.F.); 5 Department of Addiction Medicine, Grenobles Alpes University Hospital, Faculty of Medicine, Grenoble Alpes University, 38400 Grenoble, France; maurice.dematteis@ujf-grenoble.fr

**Keywords:** probability discounting, risky decision making, gambling, scoping review, sleep deprivation

## Abstract

Animals, including humans, frequently make decisions involving risk or uncertainty. Different strategies in these decisions can be advantageous depending the circumstances. Short sleep duration seems to be associated with more risky decisions in humans. Animal models for risk-based decision making can increase mechanistic understanding, but very little data is available concerning the effects of sleep. We combined primary- and meta-research to explore the relationship between sleep and risk-based decision making in animals. Our first objective was to create an overview of the available animal models for risky decision making. We performed a systematic scoping review. Our searches in Pubmed and Psychinfo retrieved 712 references, of which 235 were included. Animal models for risk-based decision making have been described for rodents, non-human primates, birds, pigs and honey-bees. We discuss task designs and model validity. Our second objective was to apply this knowledge and perform a pilot study on the effect of sleep deprivation. We trained and tested male Wistar rats on a probability discounting task; a “safe” lever always resulted in 1 reward, a “risky” lever resulted in 4 or no rewards. Rats adapted their preferences to variations in reward probabilities (*p* < 0.001), but 12 h of sleep deprivation during the light phase did not clearly alter risk preference (*p* = 0.21).

## 1. Introduction

Goal-directed behaviour is essential in daily life, and animals, including humans, frequently make decisions involving risk or uncertainty in reaching a goal. Depending on the environment and circumstances, preference for or avoidance of uncertain outcomes can be beneficial. However, risk taking can also have negative consequences and it is associated with several neurologic and psychiatric conditions, comprising attention-deficit hyperactivity disorder, frontotemporal dementia, unipolar and bipolar depression, anxiety disorders, obsessive-compulsive disorder and schizophrenia [1,2,3,4]. Furthermore, impulsive decision making could be at the base of addictive disorders with or without substance abuse (pathological gambling) [5,6,7,8,9].

Humans are generally risk-aversive when considering potential gains, and more risk-prone when facing potential losses [10]. Sleep deprivation may increase risk taking in humans. In the laboratory, sleep deprivation results in selection of riskier decks of cards in the Iowa gambling task (IGT) [11,12]. Riskier decisions were associated with shorter REM sleep duration [11]. Also, normal sleep after first task exposure seems to improve subsequent performance on the IGT [13]. Sleep deprivation has similarly been shown to affect risk-based decision making on other laboratory tasks, e.g., the Mosaic Task [14], the Columbia Card Task [14] and the Lottery Choice Task [10].

Outside the laboratory, sleep deprivation seems to increase risk taking in, e.g., alcohol and other drug use, violent/delinquent behaviour, transport risk-taking/road safety, and sexual risk taking [10,12,15,16,17]. The reason for sleep deprivation (voluntary vs. involuntary) may affect the outcome; participants with voluntary sleep loss are more risks-prone concerning driving and drinking alcohol, and they have higher disinhibition than those with insomnia and normal sleepers [17].

Decision making apparently depends on a network comprising the orbitofrontal cortex, somatosensory cortex, amygdala and striatum [3], and several neurotransmitters, comprising dopamine, serotonin, noradrenaline, acetylcholine and glutamate [4]. Further knowledge of the network and neurochemistry can result in innovative treatment for the patients suffering from impaired decision making [3], and help to limit the aversive consequences of risk taking after sleep deprivation. Animal models allow for mechanistic studies which could lead to improved insights into the neurochemistry, but up to now validated study design data are scarce, as are experimental data.

The optimal animal model and experimental design to answer a research question should be selected based on previous work. Interpretation of the results also requires awareness of the related literature. Reproducible systematic review techniques can aid evidence-based decision making on the experimental design and interpretation of the results. One review type implementing systematic methods is the scoping review [18,19], which provides an explorative analysis of the literature on a topic such as animal models for risk taking.

Behavioural tasks have been developed to test risk taking and gambling in laboratory settings. Many possible task designs have been developed for use in humans and animals. While these tasks have been reviewed before (e.g., [4,20,21,22,23,24]), we are not aware of any systematic overview. In this work, we thus describe a combined approach, consisting of a systematic scoping review (meta-research) and a primary pilot-study, to explore the relationship between sleep and risk-based decision making in animals.

The systematic scoping review answers the review question: “What are the available animal models to test risk-based decision making?” To find out if the effect of sleep deprivation on risk-based decision making in rats is comparable to that in man and might be used as a model to elucidate underlying mechanisms, we performed a pilot experiment to address the research question: “Is risk-based decision making affected by sleep deprivation in male Wistar rats?” The main purpose of this pilot study was to determine the standard deviation in risk preference at baseline, and the potential effect size of sleep deprivation. These parameters are needed to reliably estimate how many animals would be needed for a full experiment.

## 2. Results

### 2.1. Systematic Scoping Review

#### 2.1.1. Reference Flow

**Figure 1:**
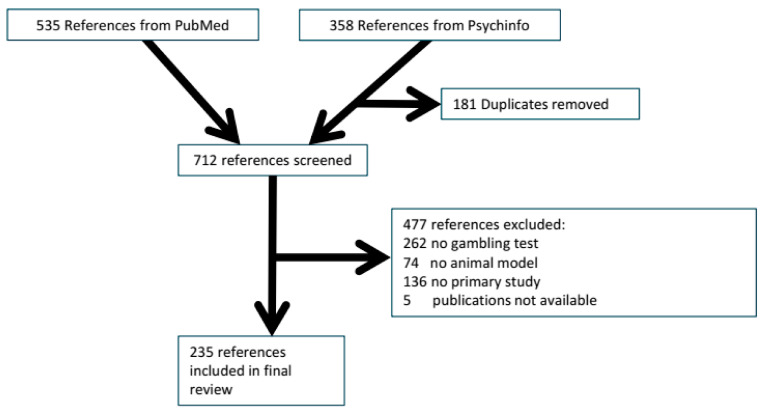
Flow scheme of references.

Our most recent search in Pubmed retrieved 535 papers, while that in Embase retrieved 358. After duplicate removal, 712 references were screened for inclusion. From these 712 references, 235 described at least one animal model for risk-based decision making and were included in our review. The reference flow and reasons for exclusion are summarised in Figure 1.

**Figure 2:**
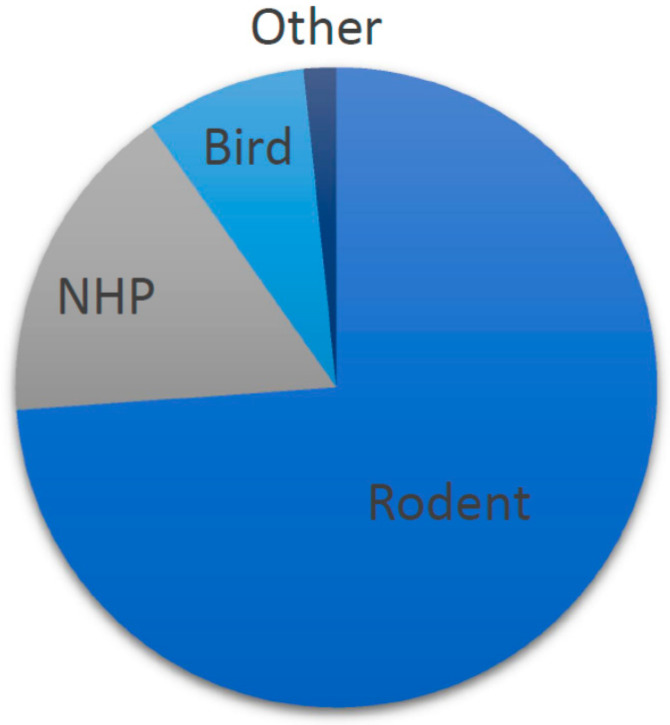
Animals used in included studies.

The 235 references described 245 risk-based decision making tasks in various species. Species comprised rodents (k = 181, rats and mice), non-human primates (NHPs) (k = 40, various species) birds (k = 20, pigeons and starlings), pigs (k = 2) and honey bees (k = 2). The distribution of the species-categories of the included studies is shown in Figure 2. Lists of the included papers are provided by species category in the Supplementary File.

The sex of the tested animals was reported for 217 of the tasks. Most tested only male animals (k = 178), k = 3 tested only female animals, and k = 36 tested both males and females. Most tests were performed in an operant cage (k = 177). Alternatives were different kinds of mazes (k = 15) and other test boxes. Testing in the home cage is not yet common (k = 7). Rewards for the animals generally comprise drinks or food; the included publications used, e.g., vegetables, crackers, chocolate, marshmallows, precision pellets, (sugar)water, fruit juices, condensed milk and milkshakes. Alternatives are direct brain stimulation [25] and (in NHPs) tokens that are exchanged for juice at a later stage [26].

#### 2.1.2. Narrative Summary of Animal Models for Risk-Based Decision Making

The included papers described highly variable task designs, with little consistency in terminology between research groups. Also, descriptions of the experiments sometimes lack sufficient detail for full evaluation and categorisation. Moreover, some tasks that are very comparable in design are conceptually quite different, which makes the categorisation of tasks disputable. Therefore, we cannot quantitatively summarise our data by overall task design. To provide an indication of the different types of tasks used, we present a narrative summary of the three main categories we observed. These categories are summarised in Table 1.

##### Probability Discounting

The majority of the included publications described some kind of probability discounting task. In these tasks, animals are given the choice between a safe and a risky option to attain reward. In general, the reward on the risky choice is larger than the reward on the safe choice, but the amount of reward can be varied for both the safe, e.g., [27,28] and the risky (see below) option. The authors used various synonyms, e.g., betting task, discrete-trial choice task, economic decision-making task, food-gambling task, pig gambling task, probabilistic choice task, rat gambling task, risk discounting task, risky choice task, risky decision task, risky decision making task, risk preference test and visual gambling task. These tasks are mainly used in rats, various species of NHPs and pigeons, but also in mice [29,30], starlings [31,32], honey bees [33,34] and pigs [35].

Both the reward size and the probability of “winning” on the risky choice can be varied. The average net gain associated with each option, and the animal’s optimal strategy, depend on these variables. For example, if the safe option always gives a small reward, while the risky option gives a reward of 4 times the size of the small reward on 50% of the trials, the risky choice is clearly advantageous. With similar reward sizes, any probability below 25% would make the certain choice the optimal strategy.

Depending on the task design, the optimal strategy can change over time, e.g., when the reward size or the probability change within or between test sessions. When altering the probabilities on probability discounting tasks, the order in which different probabilities are given may affect the outcome [36]; ascending probabilities of winning on the risky option seem to encourage risk-avoidance compared to descending probabilities, which result in more risk-prone choice behaviour.

In general, probability discounting tasks offer the animals a binary choice between safe and risky, but many variations exist. For example, there are tasks where animals have multiple risky options and one safe one (e.g., [37,38,39]). Also, in NHPs, probability discounting can be tested by first giving a small reward and letting the animal choose to keep it, or to give it back and play for a possibly larger reward (e.g., [40]). Furthermore, the safe options can vary in reward size (e.g., [41]). Fourth, while most of the probability discounting tasks are positively reinforced, some of them paired larger rewards with the risk of foot shocks (e.g., [42]).

In probability discounting tasks, risky and safe choice options can be based on discrimination of place (left/right lever/nose poke hole/arm on a maze) or distinct visual, auditory or odour stimuli. Choices can be additionally cued; different stimuli can indicate reward size and probability [26,43]. Alternatively, in NHPs, the choice can be between a safe cup and several uncertain cups with varying content, with the rewards that go into the different cups shown upfront (e.g., [44]). Also, the feed-back after the choice can be limited to the actual reward, or be additionally cued, e.g., by lights in the feeder or cue lights elsewhere. The latter is very common in the protocols used with pigeons (e.g., [45]).

The main analysed outcome measure for probability discounting tasks is the percentage or proportion risky choices. Additional measures comprised the percentage optimal choice (which depends on the reward sizes and chance level), the total amount of rewards earned, and the effect of the preceding outcome on the new choice as, e.g., proportions of win-stay and lose-switch trials.

##### Animal Versions of the Iowa Gambling Task

Besides the probability discounting tasks, animal versions of the IGT are popular. These tasks are based on the human IGT; a task in which subjects repeatedly choose between four different stacks of cards [46,47]. Each choice results in a reward or a penalty, each stack has a different average outcome. Two stacks contain cards with large rewards but also large penalties, the other two contain cards with smaller rewards but also smaller penalties. Over time, the two stacks with large rewards and penalties are not profitable, while the stacks with small reward and penalties are. This task has been adapted for animals using mazes and operant chambers [48,49]. The authors of the animal IGT-like papers also used various synonyms, e.g., gambling task, mouse gambling task, mouse Iowa gambling task, pig gambling task, primate gambling task, rat gambling task, rodent gambling task and rodent Iowa gambling task. These tasks are mainly used in rats, and to a lesser extent in mice. They have also been described for pigs [50], pigeons [45,51] and rhesus macaques [52].

The different response options (maze arms or nose poking holes in operant chambers) offer rewards of different size at different probabilities. Over time, these options are more or less profitable. Punishments/losses are either time-outs or quinine pellets. The main outcomes are the numbers/percentages/proportions of choices for each individual option, which can be pooled to choices for advantageous and disadvantageous options, and the total number/amount of rewards earned. Additionally, many authors analyse the effects of the preceding outcome on the new choice as, e.g., proportions of win-stay and lose-switch trials.

Probability discounting tasks and animal versions of Iowa-like tasks are not that different. This is nicely illustrated by a paper by Zentall et al. [45]. They perform two rather similar experiments, but the optimal choice is always rewarded in experiment 1 (thus, it had a safe option and we categorised it as probability discounting), while it was only rewarded in 75% of the trials in experiment 2 (thus, we categorised experiment 2 as IGT-like). Furthermore, tasks can be a cross-over of the two types of tasks by alternating binary choices between two uncertain outcomes (IGT-like) with binary choices with one safe outcome (probability discounting) on different trials [26,53]. Additionally, in tasks combining variable ratio (VR) and fixed ratio (FR) reward schemes, the safer option is not necessarily the optimal choice [25,54,55].

The names used by the authors do not clearly distinguish the tasks, if the authors report using the rat gambling task, they could have used either be a probabilistic discounting task or an IGT-like task.

##### The Balloon Analogue Risk Task

The last task that was repeatedly described is the balloon analogue risk task (BART). In the human task, participants can sequentially pump virtual balloons by clicking a button [56]. Virtual monetary reward is given for each pump, but if the balloon pops before participants collect their reward, all earnings for that balloon are lost.

Within our sample of the literature, an animal alternative of the BART has only been described for rats. In the rat analogue balloon task, rats are given a binary choice between two levers [57]. One of the levers is the “increase reward” lever, when this lever is pressed the prospected reward increases by one. The other lever is the “cash-out” lever, pressing it results in receiving the build-up number of rewards. The “increase reward” however has a probability of losing all rewards and starting a time-out.

The analysed outcomes are the mean number of lever presses over all or only for successful trials, the total number of rewards earned, the percentage of trials on which rats used the optimal strategy and the number of failed trials.

##### Internal and External Validity of Animal Risk-Based Decision Making Tasks

As the goal of this review was descriptive, we did not perform a formal quality assessment of the included studies. Thus, we cannot make conclusive statements on the internal validity of these models. However, it is safe to assume that the risk of bias is unclear for the majority of the included studies, as systematic reviews of animal studies consistently report the lack of reporting experimental details. Besides the standard study quality elements, in risk-based decision making studies, it is important to ward against side preferences confounding the results. Some authors explicitly mention using the same lever as the risky lever for all animals [58], others describe appropriate counterbalancing within and between animals [54,59].

Several authors have analysed the external, animal-to-human translational validity of their models by performing comparable tests in humans. Two studies compared NHPs with humans. Rivière et al., used a variant of probability discounting, where red-capped mangabeys and children could choose between 5 cups [44]. A single (striped) green cup always contained a single reward (chocolate chips for the children, cake for the mangabeys). One out of the four other, indistinguishable pink cups, contained four rewards, resulting in equivalent expected value. The authors concluded that toddlers show a strong risk preference, while monkeys do not. Perdue and Brown concluded that there is some degree of continuity between humans, Capuchin monkeys and Rhesus macaques in the desire to have choice simply for the sake of having choice on a binary choice task with one stimulus indicating one or four rewards and the other two or three [60].

Two studies compared pigeons with humans. Ludvig et al., tested pigeons and humans on formally identical choice tasks [61]. The test comprised binary choices between two out of four stimuli: safe choice small reward, risky choice large reward, safe choice large reward and risky choice small reward. They concluded that both species were more risk seeking for larger rewards than for smaller ones. McDevitt et al., tested pigeons and humans on a binary classical discounting test where the risky option was suboptimal (a large reward was only provided on 20% of the trials) [62]. They showed that both pigeons and humans can choose suboptimally, but stimuli presented during the delays affected both species differently.

Two studies compared rodents with humans on the IGT. Van den Bos et al., compared the performance of rats and mice upon introduction of the rodent IGT with typical human performance [48]. They concluded that rats, mice and humans show similar learning curves. Van Enkhuizen et al., used the standardized task with the four computerized card decks for humans. For mice, a single-session test was used where a nosepoke in one of four illuminated holes was rewarded with strawberry milkshake or punished with a time-out with contingencies comparable to the card decks in the human task. The authors concluded that the mouse IGT is similar to the human IGT for measuring risk-based decision making [63].

One study compared honey bees with humans on a binary choice probability discounting task and found comparable risk preference in both species [33].

While the studies described in this section (Internal and External Validity of Animal Risk-Based Decision Making Tasks) are interesting, and indicative of a certain amount of translational validity of these types of tasks, relatively few direct comparisons have been made. Additionally, the study designs of these studies are too heterogeneous for overall conclusions on the relevance of animal risk-based decision-making tasks for humans.

### 2.2. Pilot Experiment

We trained and tested male Wistar rats on a probability discounting task, which is fully described in Section 4.2.4. On this task, a “safe” lever always resulted in one reward; a “risky” lever resulted in four or no rewards. Probabilities of reward on the risky lever were varied between, but not within sessions. Gradual training on the task, from pressing a lever until stable task performance, is described in Section 4.2.3. We only present data from after the training period, when rats were performing at a stable level. The final task combines single choice trials, providing one of the two levers, exposing the rats to the probability and reward at each leaver, and choice trials, where both levers are present. Data from single choice trials are only analysed to verify completion, which results in adequate exposure to the probability in that session. Data from choice trials are analysed for risk preference. As a learning effect may occur at the start of a session, data are not only presented for the entire session, but also separately for the second half. Analysis of the data is detailed in Section 4.2.8.

At a reward probability of 0, the risky lever is never rewarded, and the optimal decision is the safe lever. When the probability of reward on the risky lever is 0.25, the expected total amount of reward is equal for both levers. We call this the “indifference point”. At probabilities above 0.25, consistent selection of the risky lever results in higher amounts of reward than consistent selection of the safe lever. Rats were tested at each probability level once each week, in the same order: 0.50 on Mondays, 1.00 on Tuesdays, 0.00 on Wednesdays, 0.25 on Thursdays and 0.75 on Fridays. We first analysed the preference for the risky lever at varying probabilities, to verify that rats adapt their choices towards the optimum.

We then analysed the effects of 12 h of sleep deprivation on risk-based decision making in our paradigm, using a within-subject cross-over design. Performance after sleep deprivation was compared with performance under control conditions and with the preceding undisturbed baseline and following recovery. Performance under control conditions was also compared with baseline and recovery. These tests were performed in 2 × 3 sequential weeks, and only data from the Thursday tests at the indifference point were included in these analyses.

#### 2.2.1. Variation in Risk Preference with Varying Reward Probabilities

**Figure 3:**
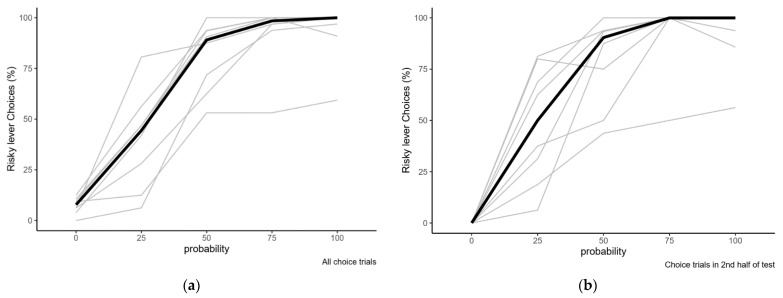
Risk preference at varying reward probabilities on the risky lever in the first baseline week. (**a**) Preference in the completed choice trials from the entire session; (**b**) Preference in the completed choice trials from the second half of the session. Individual data in grey, median values in black. Compared to the indifference point (risky lever rewarded with four pellets in 25% of the trials), rats preferred the risky lever less when it was never rewarded, and more when it was rewarded in 50–100% of the trials (statistical details provided in the text).

To verify that rats prefer the lever resulting in most rewards, we compared risk preference on the different levels of reward probability on the risky lever. Data from the first baseline week after training were analysed. There were no omissions in any of the single choice trials in the first block on any of the probabilities. Thus, all completed choice trials were included in all calculations. The overall preference for the risky lever at the different reward probabilities (individual values and median) is shown in Figure 3a. The preference for the risky lever at the different reward probabilities in the second half of the test is shown in Figure 3b. Numerical summary data are presented in Table 2.

The Friedman test showed an overall significant difference in the preference for the risky lever at different probabilities (χ^2^(4) = 29.3, *p* < 0.001). The post hoc Wilcoxon signed rank tests showed that the median preference for the risky lever was different from the indifference point (25% of trials rewarded) at all other reward probabilities (*p* < 0.01).

#### 2.2.2. Sleep Deprivation

Rats were sleep-deprived for 12 h during the light phase by means of variable forced locomotion in sleep deprivation devices (SDDs). The experiment is detailed in Section 4.2. In the sleep deprivation condition, rats were habituated to the non-moving SDDs for 2.5 days before sleep deprivation. Sleep deprivation lasted 12 h, until the Thursday test.

##### Sleep Deprivation Compared to Control Condition

**Figure 4:**
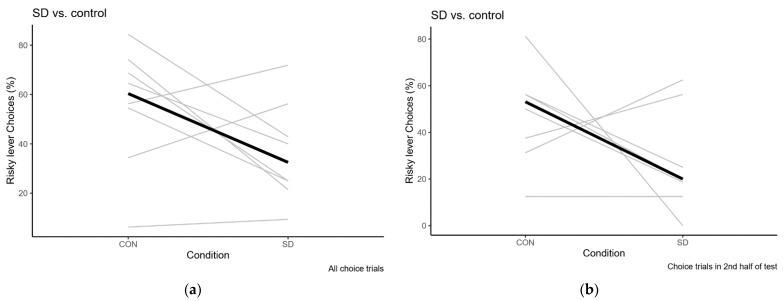
Risk preference after sleep deprivation and the control condition. (**a**) Preference in the completed choice trials from the entire session; (**b**) Preference in the completed choice trials from the second half of the session. Individual data in grey, median values in black. Please note that rat 6 is excluded from Figure 6b because there were no data in the sleep deprivation condition. In this experiment, there is no significant effect of sleep deprivation on risk preference compared to control (as detailed in the text).

In the control condition for sleep deprivation, rats were housed in non-moving SDDs. Data from the sleep deprivation condition (Table 3) were directly compared to data from the control condition (further described below, Table 4), shown in Figure 4. The Wilcoxon signed rank test showed that, in this experiment, risk preference was not significantly altered after sleep deprivation compared to control (*p* = 0.12). Of note, the post-hoc calculated power for this analysis is only 0.27, mainly due to the relatively low rank correlation in risk preference between the conditions (ρ = 0.08).

##### Sleep Deprivation Compared to Baseline

**Figure 5:**
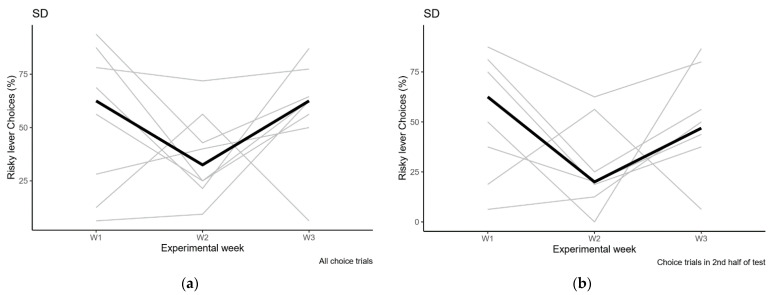
Risk preference in the sleep deprivation condition. (**a**) Preference in the completed choice trials from the entire session; (**b**) Preference in the completed choice trials from the second half of the session. Individual data in grey, median values in black. W1: baseline, W2: sleep deprivation, W3: recovery. Please note that rat 6 is excluded from Figure 5b because there were no data in the sleep deprivation condition. In this experiment, there is no significant effect of sleep deprivation on risk preference compared to baseline or recovery (as detailed in the text).

Control housing in the sleep deprivation devices might have unexpected effects on risk-based decision making, which could confound the analysis described in section Sleep Deprivation Compared to Control Condition. Therefore, data from the sleep deprivation condition, at the indifference point, were also compared with data from the week before (baseline) and the week after (recovery). Data are shown in Table 3 and Figure 5.

The Friedman test confirmed that, in this experiment, there was no significant effect of a 12 h sleep deprivation (χ^2^(2) = 3.16, *p* = 0.21) on risk preference compared to baseline and recovery.

#### 2.2.3. Control Condition

**Figure 6:**
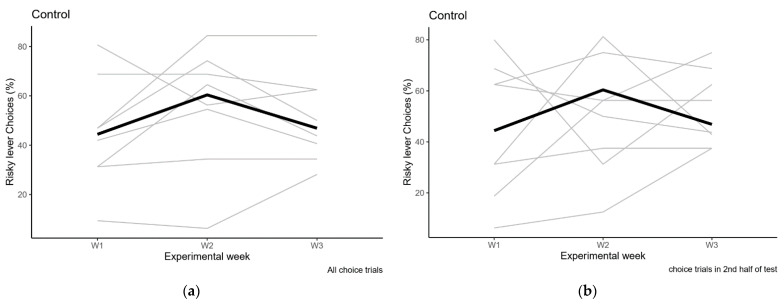
Risk preference in the control condition for sleep deprivation. (**a**) Preference in the completed choice trials from the entire session; (**b**) Preference in the completed choice trials from the second half of the session. Individual data in grey, median values in black. W1: baseline, W2: control housing in the SDDs, W3: recovery.

To visualise any potential effect of the control condition on task performance, data from the control condition Thursday, at the indifference point, were plotted with data from the week before (baseline) and the week after (recovery). Data are shown in Table 4 and Figure 6. No statistical tests were performed on these comparisons.

## 3. Discussion

The aim of systematic scoping reviews is to explore a replicable sample of literature to provide a preliminary answer to a review question [19]. In this systematic scoping review, we provide an overview of the different types of tasks used to analyse risk-based decision making in animals. We included 235 publications describing animal models for risk-based decision making in rodents, non-human primates, birds, pigs and honey bees, with unclear internal and some external validity. Most tests allow the animals to choose between options with varying chances for reward. The list of included papers is presented by species in the Supplementary Materials.

Other recent reviews have summarised the results from risk-based decision making experiments focussing on, e.g., neurochemistry [4] and anatomy [23]. We summarised the different tasks in three categories: probabilistic discounting, Iowa gambling task-like tasks and balloon analogue risk tasks. Using a relatively similar categorisation, Yates distinguished risky decision tasks where larger rewards were paired with the risk of punishment from the more standard probability discounting tasks, which were only positively, or not, reinforced [4]. We recognise the risk of punishment as a factor affecting decision making, but did not create separate summaries for positively and negatively reinforced probability discounting tasks. We preferred grouping the positive and negative reinforcements together as the risk-based decision itself may be seen as comparable (a safe small reward versus an unsure alternative). Other reviews have focussed on, e.g., models for risks versus losses [24] or gambling versus impulsivity [23].

Anselme has argued that most of the animal tasks described in this review do not address risk taking, because the occasional, unpredictable absence of reward is not a real risk [64]. The risk in positively reinforced tasks is indeed restricted to loss of reward. The idea that lacking reward is a punishment goes against the evidence that partial reinforcement in instrumental tasks actually enhances the response rates [64]. However, we think of the tasks in this paper as models for uncertainty-based decision making, even if they do not address actual risk taking. Following these semantics, token tasks and tasks comprising the risk for punishment may have wider external validity, encompassing risk-taking.

From an economic perspective, the optimal decision is the one with the highest expected value [65]. However, in decision making comprising uncertainty, there is an asymmetry between gains and losses. Humans usually tend to prefer a small certain gain over a larger more risky gain, but a large uncertain loss over a small certain loss [10]. Additionally, they often overrate low probability outcomes and devalue high probability outcomes [66]. These concepts can result in suboptimal decision making.

Compared to preceding reviews [4,20,21,22,23,24], our scoping review implemented a more thorough, replicable methodology. However, it is not a full systematic review, but a scoping review with the corresponding limitations. The main limitation is that the literature searches were not comprehensive. We are aware of a few papers that were not retrieved by our searches. For example, our searches retrieved a thesis [67], but not an original relevant publication [68] which was reprinted as chapter 2 in the thesis.

Together with our preceding observations on searching literature databases for studies on T-maze tasks [69] and activity measurements [19], this illustrates a general problem for comprehensively retrieving the literature on animal behavioural tasks via searching literature databases. This problem is caused by behavioural studies being published in journals from different fields, mainly medicine, psychology, biology and veterinary sciences, which are indexed by different databases. For full comprehensive systematic reviews of animal behavioural tasks, searching multiple databases and supplemental search strategies are thus crucial.

In the pilot experiment, rats adapted their preferences to variations in reward probabilities, showing that they learned the task and could make economic decisions and distinguish the overall expected values. This is in line with the literature (e.g., [65,70,71]).

We performed the pilot sleep deprivation experiment at the indifference point, where equal amounts of rewards were expected with consistently pressing one of the two levers, i.e., always one pellet on the safe lever or four pellets on 25% of the trials on the risky lever. Tests at the indifference point can thus be considered to reflect risk preference instead of economic decision making. In our experiment, 12 h of sleep deprivation during the light phase did not significantly alter risk preference, in two different analyses: a comparison with a control condition and one with undisturbed baseline performance. However, because of the high variability in performance within subjects and non-parametric testing, our analyses are underpowered. The graphs may suggest that individual rats respond differently to sleep deprivation: five out of eight rats decreased their risk preference after SD. While testing at the indifference point is theoretically interesting, we recommend reducing the variation in performance before further testing using this procedure, as large numbers of animals would be needed for adequately powered conclusive results.

Our scoping review identified only one other animal sleep deprivation study [72]. In mice, 24 h of sleep deprivation showed a small decrease in the percentage advantageous choices on a maze-based Iowa-like task with varying probabilities for reward and quinine pellets. The mice thus seem to increase their risk preference after sleep deprivation, while five of our rats decreased their risk preference. The different results may be explained by several factors.

First, this could be caused by differences in task design. In the maze task, there were clearly advantageous and disadvantageous options, but overall, mice selected advantageous options on only slightly over 60% of the trials. In our Skinnerbox probability discounting task, at the equivalence point there was no advantageous option. In line with this, the literature on human laboratory risk-based decision making after sleep deprivation is also inconsistent; while alterations in risk taking have been reported by several authors on several tasks [10,11,12,73,74], other risk-related tasks were not affected [73,75,76]. Full systematic reviews with meta-analyses are necessary to determine the overall effect of sleep deprivation on specific types of risk-based decision making (i.e., on specific laboratory tests) in humans.

Second, the difference in results could be caused by a species effect; rats may be more resistant to sleep deprivation than mice. Third, the difference in results could be caused by a “dose” effect; the mice were sleep deprived for 24 h, our rats for only 12 h, which may be too short to elicit an effect on risk taking. In line with this, experiments in human subjects have shown that risk taking was not affected by one night of total sleep deprivation, but altered after multiple (5 or 7) nights of sleep restriction resulting in larger cumulative sleep loss [14,77]. Another potentially relevant difference between the studies is the sleep deprivation method: platform bouncing versus forced locomotion.

This paper shows that the effect of uncertainty on decision making can be tested in several animal models. Differences between species and sexes have been observed, highlighting the importance of an appropriate experimental design. Our pilot results suggest that male Wistar rats may not change their risk preference at the indifference point after 12 h of sleep deprivation in the light phase. However, the current study design is not sufficient to draw reliable conclusions based on a realistic number of animals, the variance is too large.

At this stage, the overall evidence base for the general effects of sleep deprivation on animal risk-based decision making remains small and further studies are still necessary. Yet, future animal studies should be preceded by further analyses of human risk-based decision making after sleep deprivation.

## 4. Methods

### 4.1. Systematic Scoping Review

Our scoping review followed an informal protocol which was not externally registered; we did not expect to publish the results upon initiation of this work. We searched two literature databases for studies on animal models for gambling/ risk-based decision making: PubMed and PsychInfo. Searches comprised both terms from their internal thesauri and words in the title, abstract and keywords. Individual search terms were tested for each database, and terms that retrieved relevant literature without exploding the search with irrelevant hits were included in the final search strings. This process resulted in asymmetries between the search strings for the two databases. The resulting search strategy was reviewed by an information specialist. The search strings for gambling/ risk-based decision-making tests are provided in Table 5. These strings were combined with standard animal filters [78,79]. The first searches were performed 6 July 2017. Searches were updated on 6 August 2020.

Screening was performed by two independent reviewers for publications indexed up to 6 July 2017 (our original search date). Screening for the update (for publications after 6 July 2017) was performed by one reviewer. To be included in this review, the publication had to describe an original study, it had to describe a behavioural task relating to individual risk-based decision making/gambling and the task had to be performed by animals. Excluded were other behavioural tasks, exclusively human studies and reviews [20,24,80,81].

Clearly defining risk-based decision making and gambling is challenging. For the purpose of this review, we operationalise decision making as actively selecting one from several options [2], and we restricted to decision-making involving risk (i.e., unknown outcome). The following types of experiments (with examples) were thus excluded from this review: tasks without an active choice (e.g., single-option response tasks [82], progressive ratio tasks [83,84] or Pavlovian autoshaping [85]), delay discounting tasks without an uncertain component [86], naturalistic responses to alarm cues [87], risk taking outside the laboratory [88], time spent on open arms of an elevated maze [89,90] or parts of other devices [91,92], risk-based decision making including social components (e.g., social risk taking comparing selfish and social choices in Non-Human Primates (NHPs) [93] and collective decision making on risky or safe options in ants [94]), and self-administration studies of addiction [95]. Also excluded was the rodent slot machine task [96,97,98]. While this last task seems relevant, the choices the animals make are not chance-related; it focusses on distinguishing a win from a loss.

Data extraction was performed by a single reviewer (SvdM or CL). Extracted were the species, sex, test name according to the original authors, test setting and outcome measures related to decision making. About half of the data underwent quality control, in which no discrepancies were identified.

Species were categorised as non-human primates (NHP), rodents, birds and other. If the same test paradigm was used in multiple species from the same category, it was included in the analyses as one test. If the same test paradigm was used for, e.g., non-human primates and rodents, it was included twice. The unit of measurement for the analyses was the risk-based decision-making model; if different tests or species from different categories were described in a single publication, these were separately included in the tables and analyses.

### 4.2. Pilot Experiment

#### 4.2.1. Animals and Husbandry

The pilot experiment was performed in 8 male Wistar rats (Harlan, Horst, The Netherlands; weight on arrival 250–300 g, estimated age 7–8 weeks), as was common practice at the time (the experiment was performed in 2008). We strongly recommend including both male and female animals for any future experiment, particularly in this field, as sex differences in risk-based decision making have been observed in animals and humans [73,99,100].

The animals were housed in groups of four rats in in type-IV Makrolon (polycarbonate) cages (60 × 38 × 20 cm) with stainless steel wire covers holding a water bottle (tap water), and during the habituation phase standard laboratory chow. Home cages contained standard bedding and a shelter (12 cm high, 25 cm PVC tubing cut through lengthwise) for cage enrichment.

The homecages were placed in a light-controlled cabinet with an inverted light-dark cycle of 12:12 h, lights on at 23:15. The cabinet maintained the temperature (22.5 °C ± 1.5) and humidity (31.6% ± 8.04) at a stable level. On Fridays, cages were replaced by clean ones, always transferring one hand of bedding from the old cage to the new one to increase familiar odours. The animal facility was conventional (i.e., non-SPF). Rats were observed in their home cage each day to monitor visible indicators of lack of wellbeing (e.g., piloerection, poor grooming, lethargy, discharge from nose or ears). Humane endpoints were not explicitly defined.

Adaptation after arrival lasted 1 week, during which period the animals had free access to standard laboratory rat chow and bottled tap water. Animals were handled and weighed daily. Upon first handling by an experimenter, they were numbered with waterproof marker on the tail, animal number reflecting the order of catching them at that time (1–8). Two days prior to training, daily food was restricted to 15 g per rat (60 g per home cage) per day. On test days, daily food was adjusted to correct for the amount of food obtained in the test. Access to water remained ad libitum throughout the experiment.

All experimental protocols were approved by the ethical review committee (DEC; Dier-Experimenten Commissie, P_NIN2007-45 with an addendum) and were in accordance with the Dutch law (“Wet op de dierproeven”) and European guidelines (Guideline 86/609/EEC) that were applicable at the time of the experiment (2008).

#### 4.2.2. General Behavioural Test Procedures

Behavioural sessions were performed only on weekdays, excluding national holidays. Experiments testing the effects of sleep deprivation were planned in three sequential weeks (5 days/week) without national holidays. For each test session, rats were transported from the light-controlled cabinet to the skinner boxes within the same room. Behavioural sessions were started as soon as all the rats had been placed in their individual skinner box (30 cm × 30 cm × 30 cm aluminium Skinner boxes, Med Associates Inc., Fairfax, VT, USA). Skinner boxes were controlled by MED-PC IV software (Med Associates Inc., Fairfax, VT, USA).

The first test of the day was always performed as soon as possible after the onset of the dark phase at 11:15. During the autoshaping phase, a remedial second test could be added for some or all rats half a day later. After shaping, a single session was performed each day at the onset of the dark phase at 11:15.

#### 4.2.3. Training

The rats underwent a training program as summarised in Table 6.

In autoshaping, the house light was lit throughout, and at random intervals either the left or the right lever was presented, accompanied by its corresponding lever light. Each lever was presented until it was pressed (FR1) or 150 s had passed without a press (omission). Upon pressing, the lever was retracted, the lever light was turned off, the feeder light was turned on and one pellet was delivered in the feeder. An omission was followed by retraction of the lever and 5 s of darkness.

For one rat (rat #7), the autoshaping was supplemented with hand shaping, because it was not learning as fast as the others. In hand shaping, an experimenter observed the rat and reinforced behaviours increasingly close to lever pressing by overruling the skinner box with a remote control. At first, even orientation towards the lever was rewarded. When the rat consistently oriented towards the lever for reward, active approaches were rewarded, then lever contacts, until the rat started pressing the lever. Shaping was performed until all animals responded in 90% of the trials (5–8 sessions).

Operant responses were progressively increased to FR4 in one session, to prevent later choice trials being affected by accidental lever presses. The progressive ratio session started with 10 trials on FR1, followed by 10 at FR2, 10 at FR3 and 34 trials at FR4. In the next phase, rats had to initiate the trials with a nosepoke in the feeder, signalized by the feeder light. Active trial initiation positions the rat in the middle of the skinner box, decreasing effects of potential side bias.

#### 4.2.4. Probability Discounting Choice Task

**Figure 7:**
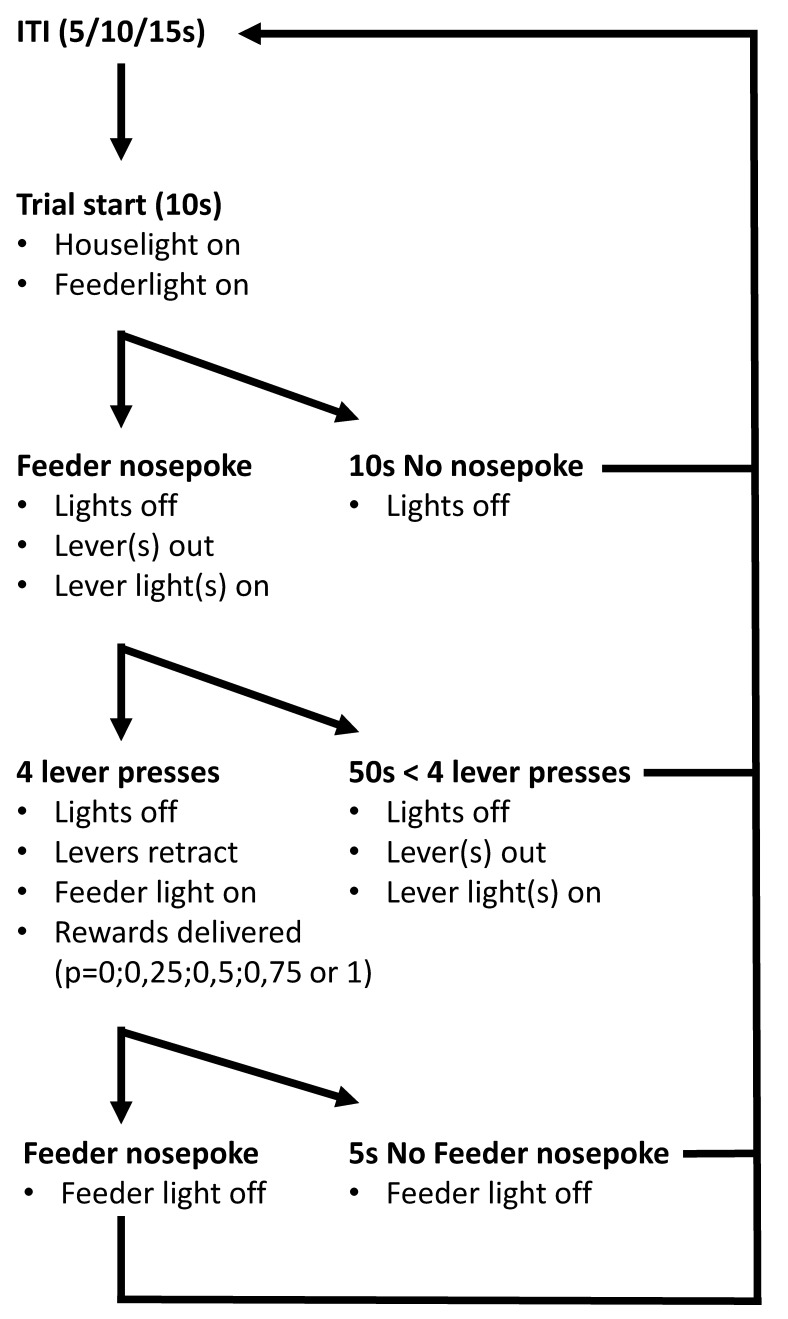
Flow chart of a trial on the probability discounting task (ITI: Intertrial interval).

We modelled our final task on a probability discounting task developed by colleagues elsewhere [101]. Our final task consisted of 8 blocks of 12 trials (total: 96 trials). The first eight trials of each block were single choice trials, only one of the two levers was presented (four times left and four times right). In the single choice trials, the rats sampled the chance and reward contingencies for that test on each lever. The last four trials of each block were choice trials, rats could choose between the two levers. A flow scheme of a choice trial is provided in Figure 7.

In each session, one of the levers was the “safe” lever, it always offered one pellet (probability = 1.00). The other lever was the “risky” lever, which offered four pellets, but with variable probability levels (0.00, 0.25, 0.50, 0.75 or 1.00). The probability on the risky lever was constant over the session. The designated risky lever was the same over a session, but varied between sessions. The animals were tested on the different probability levels in 38 sessions (each probability level at most once each week, order varied) to reach stable response levels. Data from the training period are not shown.

#### 4.2.5. Risk Preference at Different Probability Levels

For the remainder of the experiment, rats were tested at each probability level once each week, in the same order. The probability of reward on the risky lever was 0.50 on Mondays, 1.00 on Tuesdays, 0.00 on Wednesdays, 0.25 on Thursdays and 0.75 on Fridays. From this phase onwards, the risky lever remained on the same side for an individual rat, but balanced between rats (left for odd-numbered rats, right for even-numbered rats).

An exploratory analysis was performed to determine the risk preference on the different probability levels in the week before the first sleep deprivation.

#### 4.2.6. Sleep Deprivation

Sleep deprivation was tested in two sets of three sequential experimental weeks (15 sessions); a baseline week, a week with a single exposure to 12 h of sleep deprivation during the rest (light) phase or control condition before the Thursday test, and a recovery week. Comparisons were only made between Thursday sessions at the indifference point, i.e., at the 25% probability of reward at the risky lever. By consequently responding on either the safe (one pellet each trial) or the risky (four pellets once in every four trials) lever, the rat can obtain the same amount of reinforcement.

The effect of sleep deprivation was tested in all rats in a counterbalanced manner; rats 1 and 2 (cage A) and rats 5 and 6 (cage B) were first tested in the control condition, and 4 weeks later in the sleep deprivation condition. For rats 3 and 4 (cage A) and rats 7 and 8 (cage B), the test sequence was reversed. There was no apparent effect of test order on the outcome (not formally analysed).

Sleep deprivation was realised by variable forced locomotion, according to a previously described and validated protocol [102]. In short, the sleep deprivation devices consist of an upright drum, which can turn in both directions, with an immobile transparent wall in the middle. One rat was housed on each side of the wall; communication with the other rat was possible via holes in the wall. Food (restricted) and water (ad libitum) were available in the devices as in the home cage.

The rats were habituated to the SDDs from Monday, after the probability discounting test, onwards, and only taken out for daily testing. During these periods, the SDDs did not turn; rats were free to sleep as they pleased. Rats were exposed to 12 h of sleep deprivation during the light phase, or left undisturbed, in the 12 h preceding their Thursday behavioural session. Locomotor activity was measured during the sleep deprivation period, confirming that the sleep-deprived rats were active throughout while activity levels in control rats were lower (data not shown).

#### 4.2.7. Experimental Design

No protocol was preregistered for this experiment as no registries were available at the time. The experimental unit was the individual animal. The sample size for this pilot experiment of a novel task was based on other behavioural experiments from our group, without a formal a priori power calculation. All animals were included in all statistical analyses.

Our experimental design did not comprise randomisation and blinding. As adequate blinding for sleep deprivation is not possible, sequence predictability is not a concern. We thus generally focus sleep deprivation experimental designs on appropriate counterbalancing, to prevent randomisation failure affecting the outcomes. For this experiment, we used a within-subject design, counterbalancing the test order between animals, home cage/housing group, skinnerbox and SDD.

#### 4.2.8. Data Analysis

We only present data from our main outcome measure: risk preference. Risk preference was calculated as the percentage of choices for the risky lever on completed free choice trials. Completion of all single choice trials in the first block of each test was verified to ensure adequate sampling (i.e., all single choice trials completed) of the probability and reward size on both levers. Inadequate sampling only occurred once in the control for sleep deprivation condition (see below). We show the preference for the risky lever separately for the entire sessions (32 choice trials) and for the last four blocks (16 choice trials) in each session. Data are presented as median and range (minimum–maximum). Graphs show medians (in black) and individual animal data (in grey). Statistical analyses were only performed for the entire sessions.

We started with an exploratory analysis of baseline choice behaviour on the various probabilities during the week before the first sleep deprivation (i.e., the first baseline week) with a Friedman test. Next, we analysed the effect of sleep deprivation within subjects for all 8 rats in two manners. We compared risk preference after sleep deprivation with risk preference after control housing in the sleep deprivation devices with a Wilcoxon signed rank test. Risk preference after sleep deprivation was also compared to both baseline (1 week earlier on the same day and at the indifference probability level) and recovery (1 week later) with a Friedman test. As no effect was found, no further analyses were performed, and the control condition was not statistically compared to the preceding and following week.

As we cannot assume a normal distribution with percentages based on at most 32 observations, we used non-parametric tests. Our data meet the assumptions of the Friedman and Wilcoxon signed rank tests: repeated measures within independent subjects. For the Friedman test comparing risk preference on the various probabilities, post-hoc Wilcoxon signed-rank tests were performed comparing the other probabilities to the indifference point.

Within the sleep deprivation experiment, only one rat (rat 1) omitted one single choice trial (the fifth, on the risky lever) within the first block of the test in the control condition. This rat did not omit any single choice trials in the second block, so all further data were included and percentages for this session for this rat were based on the 28 choice trials from block 2 onwards. Another rat (rat 6) started omitting all trials from the 31st trial onwards after sleep deprivation. As the activity levels measured in the skinnerbox at this time were low, we suspect that this rat went to sleep. For this rat in this session, overall data are shown based on the completed choice trials, data from the 2nd half of the test are left out of the graphs.

Data were copied from the skinnerbox output (MedPC files) to Excel for pre-processing, and analysed in R version 3.6.3 (29 February 2020)—“Holding the Windsock” via RStudio [103]. Data were imported using the readxl package [104], organised and analysed using the dplyr package [105], and visualised using the ggplot2 package [106]. A two-tailed post-hoc power calculation was performed in G*Power 3.1, using α = 0.05 and actual values for the comparison between sleep deprivation and control.

## Figures and Tables

**Table 1:** Summary of task categories.

Task Category	Animal Species Tested	Brief Description	Comparable Tests in Humans
Probability Discounting (PD)	Rats, NHPs, Pigeons, Mice, Starlings, Bees, Pigs.	Choices between safe and risky options.	Human PD tests; tests with safe and risky options.
Iowa Gambling Task (IGT)	Rats, Mice, Pigs, Pigeons, Macaques.	Repetitive choices between options that are on average advantageous or disadvantageous, with a risk for loss/punishment at each option.	Human IGT, picking cards that will result in gains or losses from 4 card decks. Two decks are on average advantageous, the others are on average disadvantageous.
Balloon Analogue Risk Task (BART)	Rats.	Pressing on one of two levers increases the size of a reward, but at a risk of losing everything. Pressing the other lever results in receiving the build-up reward.	Human BART; pressing a button to inflate a virtual balloon at the risk of popping it, or another button to cash out a reward proportional to the balloon size.

NHP: Non-Human Primate.

**Table 2:** Median (minimum–maximum) risk preference at varying reward probabilities.

Probability of Reward at Risky Lever	Overall % Risky Choice	Risky Choices in 2nd Half of Session
0.00	7.8 (0–12.5)	0 (0–0)
0.25	44.4 (6.3–80.6)	50 (6.3–81.3)
0.50	89.1 (53.1–100)	90.4 (43.8–100)
0.75	98.4 (53.1–100)	100 (50–100)
1.00	100 (59.4–100)	100 (56.3–100)

**Table 3:** Median (minimum–maximum) risk preference in the sleep deprivation condition.

Test	Overall % Risky Choice	Risky Choices in 2nd Half of Session
Baseline	62.5 (6.3–93.8)	62.5 (6.3–93.8)
Sleep Deprivation	32.5 (9.4–71.9)	20 (0–62.5)
Recovery	62.5 (6.3–87.1)	46.9 (6.3–86.7)

**Table 4:** Median (minimum–maximum) risk preference in the control condition.

Test	Overall % Risky Choice	Risky Choices in 2nd Half of Session
Baseline	44.4 (9.4–80.6)	46.9 (6.3–80)
Control housing in SDDs	60.4 (6.3–84.4)	53.1 (12.5–81.3)
Recovery	46.9 (28.1–84.4)	50 (37.5–75)

**Table 5:** Search strings for risk tasks.

Database	Last Update	Risk Task Search String
PubMed (legacy)	6 August 2020	((risk taking [mesh] OR risk taking [tiab] OR risk preference [tiab] OR risk-based [tiab] OR risk tolerance [tiab]) AND (decision making [mesh] OR decision making [tiab])) OR (choice behavior [majr] AND risk [tiab])
Psychinfo	10 August 2020	((Risk taking/OR gambling/OR (risk taking OR risk preference OR risk-based OR risk tolerance).mp.) AND ((decision making/OR decision making.mp.) OR (choice behavior/AND risk.mp.)))

**Table 6:** Training program.

Phase	Task	Session Length	FR	# Sessions
Autoshaping	Press lever	60 min or 64 trials	1	5–8
Progressive ratio	Press lever at FR	64 trials	1–4	1
Trial initiation	NP for trial start	64 trials	4	7
Stable risk preference	Final task	96 trials	4	38

FR: Fixed ratio (# leverpresses to reward). NP: Nosepoke.

## Data Availability

All individual animal data presented in this paper are visually available from the figures. Numerical summary data are available in the tables. Individual animal numerical data will be made available on reasonable request from the corresponding author.

## Supplementary Materials

The following are available online at https://www.mdpi.com/2624-5175/3/1/3/s1, Table S1: Publications on gambling tasks in NHPs, Table S2: Publications on gambling tasks in rodents, Table S3: Publications on gambling tasks in birds, Table S4: Publications on gambling tasks in other species.

## Abbreviations

BART	Balloon Analog Risk Task
FR	Fixed Ratio
IGT	Iowa Gambling Task
ITI	InterTrial Interval
NHP	Non-Human Primate
NP	NosePoke
SDD	Sleep Deprivation Device
VR	Variable Ratio

## References

[1] Mowinckel A.M., Pedersen M.L., Eilertsen E., Biele G. (2015). A meta-analysis of decision-making and attention in adults with ADHD. J. Atten. Disord..

[2] Paulus M.P. (2007). Decision-making dysfunctions in psychiatry—Altered homeostatic processing?. Science.

[3] Rahman S., Sahakian B.J., Cardinal R.N., Rogers R., Robbins T. (2001). Decision making and neuropsychiatry. Trends Cogn. Sci..

[4] Yates J.R. (2019). Examining the neurochemical underpinnings of animal models of risky choice: Methodological and analytic considerations. Exp. Clin. Psychopharmacol..

[5] Ioannidis K., Hook R., Wickham K., Grant J.E., Chamberlain S.R. (2019). Impulsivity in Gambling Disorder and problem gambling: A meta-analysis. Neuropsychopharmacol. Off. Publ. Am. Coll. Neuropsychopharmacol..

[6] Kyonka E.G.E., Schutte N.S. (2018). Probability discounting and gambling: A meta-analysis. Addiction.

[7] Buchanan T.W., McMullin S.D., Mulhauser K., Weinstock J., Weller J.A. (2020). Diurnal cortisol and decision making under risk in problem gambling. Psychol. Addict. Behav..

[8] Kraplin A., Dshemuchadse M., Behrendt S., Scherbaum S., Goschke T., Buhringer G. (2014). Dysfunctional decision-making in pathological gambling: Pattern specificity and the role of impulsivity. Psychiatry Res..

[9] Chen S., Yang P., Chen T., Su H., Jiang H., Zhao M. (2020). Risky decision-making in individuals with substance use disorder: A meta-analysis and meta-regression review. Psychopharmacology.

[10] McKenna B.S., Dickinson D.L., Orff H.J., Drummond S.P. (2007). The effects of one night of sleep deprivation on known-risk and ambiguous-risk decisions. J. Sleep Res..

[11] Brunet J.F., McNeil J., Doucet E., Forest G. (2020). The association between REM sleep and decision-making: Supporting evidences. Physiol. Behav..

[12] Killgore W.D., Balkin T.J., Wesensten N.J. (2006). Impaired decision making following 49 h of sleep deprivation. J. Sleep Res..

[13] Peng X.R., Liu Y.R., Fan D.Q., Lei X., Liu Q.Y., Yu J. (2020). Deciphering Age Differences in Experience-Based Decision-Making: The Role of Sleep. Nat. Sci. Sleep.

[14] Salfi F., Lauriola M., Tempesta D., Calanna P., Socci V., De Gennaro L., Ferrara M. (2020). Effects of Total and Partial Sleep Deprivation on Reflection Impulsivity and Risk-Taking in Deliberative Decision-Making. Nat. Sci. Sleep.

[15] Short M.A., Weber N. (2018). Sleep duration and risk-taking in adolescents: A systematic review and meta-analysis. Sleep Med. Rev..

[16] Millar B.M., Parsons J.T., Redline S., Duncan D.T. (2019). What’s Sleep Got to Do with It?: Sleep Health and Sexual Risk-Taking Among Men Who have Sex with Men. AIDS Behav..

[17] Rusnac N., Spitzenstetter F., Tassi P. (2019). Chronic sleep loss and risk-taking behavior: Does the origin of sleep loss matter?. Behav. Sleep Med..

[18] Arksey H., O’Malley L. (2005). Scoping studies: Towards a methodological framework. Int. J. Soc. Res. Meth..

[19] Leenaars C., Tsaioun K., Stafleu F., Rooney K., Meijboom F., Ritskes-Hoitinga M., Bleich A. (2020). Reviewing the animal literature: How to describe and choose between different types of literature reviews. Lab. Anim..

[20] Vonder Haar C. (2020). Challenges and opportunities in animal models of gambling-like behavior. Curr. Opin. Behav. Sci..

[21] Anselme P. (2013). Dopamine, motivation, and the evolutionary significance of gambling-like behaviour. Behav. Brain Res..

[22] Bailey M.R., Simpson E.H., Balsam P.D. (2016). Neural substrates underlying effort, time, and risk-based decision making in motivated behavior. Neurobiol. Learn. Mem..

[23] Barrus M.M., Winstanley C.A. (2017). Preclinical models and neurocircuitry of gambling and impulsive behavior. Curr. Opin. Behav. Sci..

[24] Freeland C.M., Knes A.S., Robinson M.J.F. (2020). Translating concepts of risk and loss in rodent models of gambling and the limitations for clinical applications. Curr. Opin. Behav. Sci..

[25] Persons A.L., Tedford S.E., Celeste T. (2017). Mirtazapine and ketanserin alter preference for gambling-like schedules of reinforcement in rats. Prog. Neuro-Psychopharmacol. Biol. Psychiatry.

[26] Farashahi S., Azab H., Hayden B., Soltani A. (2018). On the Flexibility of Basic Risk Attitudes in Monkeys. J. Neurosci. Off. J. Soc. Neurosci..

[27] Ishii H., Onodera M., Ohara S., Tsutsui K.I., Iijima T. (2018). Sex Differences in Risk Preference and c-Fos Expression in Paraventricular Thalamic Nucleus of Rats During Gambling Task. Front. Behav. Neurosci..

[28] Ito M., Takatsuru S., Saeki D. (2000). Choice between constant and variable alternatives by rats: Effects of different reinforcer amounts and energy budgets. J. Exp. Anal. Behav..

[29] Leblond M., Fan D., Brynildsen J.K., Yin H.H. (2011). Motivational state and reward content determine choice behavior under risk in mice. PLoS ONE.

[30] Parker J.G., Wanat M.J., Soden M.E., Ahmad K., Zweifel L.S., Bamford N.S., Palmiter R.D. (2011). Attenuating GABA(A) receptor signaling in dopamine neurons selectively enhances reward learning and alters risk preference in mice. J. Neurosci. Off. J. Soc. Neurosci..

[31] Aw J., Monteiro T., Vasconcelos M., Kacelnik A. (2012). Cognitive mechanisms of risky choice: Is there an evaluation cost?. Behav. Process..

[32] Shapiro M.S., Schuck-Paim C., Kacelnik A. (2012). Risk sensitivity for amounts of and delay to rewards: Adaptation for uncertainty or by-product of reward rate maximising?. Behav. Process..

[33] Shafir S., Reich T., Tsur E., Erev I., Lotem A. (2008). Perceptual accuracy and conflicting effects of certainty on risk-taking behaviour. Nature.

[34] Drezner-Levy T., Shafir S. (2007). Parameters of variable reward distributions that affect risk sensitivity of honey bees. J. Exp. Biol..

[35] Murphy E., Kraak L., van den Broek J., Nordquist R.E., van der Staay F.J. (2015). Decision-making under risk and ambiguity in low-birth-weight pigs. Anim. Cogn..

[36] Onge J.R., Ahn S., Phillips A.G., Floresco S.B. (2012). Dynamic fluctuations in dopamine efflux in the prefrontal cortex and nucleus accumbens during risk-based decision making. J. Neurosci..

[37] Haun D.B., Nawroth C., Call J. (2011). Great apes’ risk-taking strategies in a decision making task. PLoS ONE.

[38] Hayden B.Y., Heilbronner S.R., Nair A.C., Platt M.L. (2008). Cognitive influences on risk-seeking by rhesus macaques. Judgm. Decis. Mak..

[39] Morgado P., Marques F., Silva M.B., Sousa N., Cerqueira J.J. (2014). A novel risk-based decision-making paradigm. Front. Behav. Neurosci..

[40] Pele M., Broihanne M., Thierry B., Call J., Dufour V. (2014). To bet or not to bet? Decision-making under risk in non-human primates. J. Risk Uncertain..

[41] Rosati A.G., Hare B. (2013). Chimpanzees and bonobos exhibit emotional responses to decision outcomes. PLoS ONE.

[42] Shimp K.G., Mitchell M.R., Beas B., Bizon J.L., Setlow B. (2015). Affective and cognitive mechanisms of risky decision making. Neurobiol. Learn. Mem..

[43] Constantinople C.M., Piet A.T., Brody C.D. (2019). An Analysis of Decision under Risk in Rats. Curr. Biol. CB.

[44] Riviere J., Stomp M., Augustin E., Lemasson A., Blois-Heulin C. (2018). Decision-making under risk of gain in young children and mangabey monkeys. Dev. Psychobiol..

[45] Zentall T.R., Andrews D.M., Case J.P. (2017). Prior commitment: Its effect on suboptimal choice in a gambling-like task. Behav. Process..

[46] Bechara A., Damasio H., Tranel D., Damasio A.R. (1997). Deciding advantageously before knowing the advantageous strategy. Science.

[47] Bechara A., Damasio A.R., Damasio H., Anderson S.W. (1994). Insensitivity to future consequences following damage to human prefrontal cortex. Cognition.

[48] van den Bos R., Lasthuis W., den Heijer E., van der Harst J., Spruijt B. (2006). Toward a rodent model of the Iowa gambling task. Behav. Res. Methods.

[49] de Visser L., Homberg J.R., Mitsogiannis M., Zeeb F.D., Rivalan M., Fitoussi A., Galhardo V., van den Bos R., Winstanley C.A., Dellu-Hagedorn F. (2011). Rodent versions of the iowa gambling task: Opportunities and challenges for the understanding of decision-making. Front. Neurosci..

[50] van der Staay F., van Zutphen J.A., de Ridder M.M., Nordquist R.E. (2017). Effects of environmental enrichment on decision-making behavior in pigs. Appl. Anim. Behav. Sci..

[51] Laude J.R., Pattison K.F., Zentall T.R. (2012). Hungry pigeons make suboptimal choices, less hungry pigeons do not. Psychon. Bull. Rev..

[52] Strait C.E., Hayden B.Y. (2013). Preference patterns for skewed gambles in rhesus monkeys. Biol. Lett..

[53] Heilbronner S.R., Hayden B.Y. (2016). The description-experience gap in risky choice in nonhuman primates. Psychon. Bull. Rev..

[54] Johnson P.S., Madden G.J., Brewer A.T., Pinkston J.W., Fowler S.C. (2011). Effects of acute pramipexole on preference for gambling-like schedules of reinforcement in rats. Psychopharmacology.

[55] Goldshmidt J.N. (1998). Risk taking and resource scarcity: An integrative approach to foraging problems. Diss. Abstr. Int. Sect. B Sci. Eng..

[56] Lauriola M., Panno A., Levin I.P., Lejuez C.W. (2014). Individual Differences in Risky Decision Making: A Meta-analysis of Sensation Seeking and Impulsivity with the Balloon Analogue Risk Task. J. Behav. Dec. Mak..

[57] Tan D., Vyas A. (2016). Toxoplasma gondii infection and testosterone congruently increase tolerance of male rats for risk of reward forfeiture. Horm. Behav..

[58] Leblond M., Sukharnikova T., Yu C., Rossi M.A., Yin H.H. (2014). The role of pedunculopontine nucleus in choice behavior under risk. Eur. J. Neurosci..

[59] Ozga-Hess J.E., Anderson K.G. (2019). Differential effects of d-amphetamine and atomoxetine on risk-based decision making of Lewis and Fischer 344 rats. Behav. Pharmacol..

[60] Perdue B.M., Brown E.R. (2018). Irrational choice behavior in human and nonhuman primates. Anim. Cogn..

[61] Ludvig E.A., Madan C.R., Pisklak J.M., Spetch M.L. (2014). Reward context determines risky choice in pigeons and humans. Biol. Lett..

[62] McDevitt M.A., Diller J.W., Pietrzykowski M.O. (2019). Human and pigeon suboptimal choice. Learn. Behav..

[63] van Enkhuizen J., Henry B.L., Minassian A., Perry W., Milienne-Petiot M., Higa K.K., Geyer M.A., Young J.W. (2014). Reduced dopamine transporter functioning induces high-reward risk-preference consistent with bipolar disorder. Neuropsychopharmacol. Off. Publ. Am. Coll. Neuropsychopharmacol..

[64] Anselme P. (2015). Does reward unpredictability reflect risk?. Behav. Brain Res..

[65] Yang J.H., Cheng C.P., Liao R.M. (2018). Effects of d-amphetamine on risk choice in rats depend on the manner in which the expected reward value is varied. Pharmacol. Biochem. Behav..

[66] Stauffer W.R., Lak A., Bossaerts P., Schultz W. (2015). Economic choices reveal probability distortion in macaque monkeys. J. Neurosci. Off. J. Soc. Neurosci..

[67] Tryon V. (2018). Investigating the Contributions of Hippocampal Memory and Reward Valuation Systems to Cost-Benefit Decision Making. Diss. Abstr. Int. Sect. B Sci. Eng..

[68] Tryon V.L., Penner M.R., Heide S.W., King H.O., Larkin J., Mizumori S.J.Y. (2017). Hippocampal neural activity reflects the economy of choices during goaldirected navigation. Hippocampus.

[69] Leenaars C.H., Pels E.G.M., Joosten R.N., Ritskes-Hoitinga M. (2019). Wistar rats do not show preference for either of two commonly used nutritionally sound food rewards in a T-maze. J. Vet. Behav..

[70] Westbrook S.R., Hankosky E.R., Dwyer M.R., Gulley J.M. (2018). Age and sex differences in behavioral flexibility, sensitivity to reward value, and risky decision-making. Behav. Neurosci..

[71] Zoratto F., Laviola G., Adriani W. (2016). The subjective value of probabilistic outcomes: Impact of reward magnitude on choice with uncertain rewards in rats. Neurosci. Lett..

[72] Pittaras E., Callebert J., Dorey R., Chennaoui M., Granon S., Rabat A. (2018). Mouse Gambling Task reveals differential effects of acute sleep debt on decision-making and associated neurochemical changes. Sleep.

[73] Acheson A., Richards J.B., de Wit H. (2007). Effects of sleep deprivation on impulsive behaviors in men and women. Physiol. Behav..

[74] Killgore W.D., Grugle N.L., Balkin T.J. (2012). Gambling when sleep deprived: Don’t bet on stimulants. Chronobiol. Int..

[75] Menz M.M., Buchel C., Peters J. (2012). Sleep deprivation is associated with attenuated parametric valuation and control signals in the midbrain during value-based decision making. J. Neurosci. Off. J. Soc. Neurosci..

[76] Venkatraman V., Chuah Y.M., Huettel S.A., Chee M.W. (2007). Sleep deprivation elevates expectation of gains and attenuates response to losses following risky decisions. Sleep.

[77] Maric A., Montvai E., Werth E., Storz M., Leemann J., Weissengruber S., Ruff C.C., Huber R., Poryazova R., Baumann C.R. (2017). Insufficient sleep: Enhanced risk-seeking relates to low local sleep intensity. Ann. Neurol..

[78] de Vries R.B., Hooijmans C.R., Tillema A., Leenaars M., Ritskes-Hoitinga M. (2014). Updated version of the Embase search filter for animal studies. Lab. Anim..

[79] Hooijmans C.R., Tillema A., Leenaars M., Ritskes-Hoitinga M. (2010). Enhancing search efficiency by means of a search filter for finding all studies on animal experimentation in PubMed. Lab. Anim..

[80] Hart E.E., Izquierdo A. (2019). Quantity versus quality: Convergent findings in effort-based choice tasks. Behav. Process..

[81] Houston A.I., Malhotra G. (2019). Mechanistic models must link the field and the lab. Behav. Brain Sci..

[82] Fujimoto A., Minamimoto T. (2019). Trait and State-Dependent Risk Attitude of Monkeys Measured in a Single-Option Response Task. Front. Neurosci..

[83] Hart E.E., Gerson J.O., Izquierdo A. (2018). Persistent effect of withdrawal from intravenous methamphetamine self-administration on brain activation and behavioral economic indices involving an effort cost. Neuropharmacology.

[84] Hellberg S.N., Levit J.D., Robinson M.J.F. (2018). Under the influence: Effects of adolescent ethanol exposure and anxiety on motivation for uncertain gambling-like cues in male and female rats. Behav. Brain Res..

[85] Robinson M.J.F., Clibanoff C., Freeland C.M., Knes A.S., Cote J.R., Russell T.I. (2019). Distinguishing between predictive and incentive value of uncertain gambling-like cues in a Pavlovian autoshaping task. Behav. Brain Res..

[86] Linsenbardt D.N., Smoker M.P., Janetsian-Fritz S.S., Lapish C.C. (2017). Impulsivity in rodents with a genetic predisposition for excessive alcohol consumption is associated with a lack of a prospective strategy. Cogn. Affect. Behav. Neurosci..

[87] Darby N.A., McGhee K.E. (2019). Boldness is affected by recent experience with predation cues and body size in mosquitofish. Behav. Process..

[88] O’Reilly A.O., Hofmann G., Mettke-Hofmann C. (2019). Gouldian finches are followers with black-headed females taking the lead. PLoS ONE.

[89] Cheng W.H., Martens K.M., Bashir A., Cheung H., Stukas S., Gibbs E., Namjoshi D.R., Button E.B., Wilkinson A., Barron C.J. (2019). CHIMERA repetitive mild traumatic brain injury induces chronic behavioural and neuropathological phenotypes in wild-type and APP/PS1 mice. Alzheimer’s Res. Ther..

[90] Faure A., Zoratto F., Chirico D., Romano E., Mancinelli R., Saso L., Callebert J., Laviola G., Granon S., Adriani W. (2019). Reduced adolescent risk-assessment and lower nicotinic beta-2 expression in rats exposed to nicotine through lactation by forcedly drinking dams. Neuroscience.

[91] Munoz-Villegas P., Rodriguez V.M., Giordano M., Juarez J. (2017). Risk-taking, locomotor activity and dopamine levels in the nucleus accumbens and medial prefrontal cortex in male rats treated prenatally with alcohol. Pharmacol. Biochem. Behav..

[92] Hebert M.A., Ardid D., Henrie J.A., Tamashiro K., Blanchard D.C., Blanchard R.J. (1999). Amygdala lesions produce analgesia in a novel, ethologically relevant acute pain test. Physiol. Behav..

[93] Calcutt S.E., Proctor D., Berman S.M., de Waal F.B.M. (2019). Chimpanzees (Pan troglodytes) Are More Averse to Social Than Nonsocial Risk. Psychol. Sci..

[94] Hubner C., Czaczkes T.J. (2017). Risk preference during collective decision making: Ant colonies make risk-indifferent collective choices. Anim. Behav..

[95] Nentwig T.B., Starr E.M., Chandler L.J., Glover E.J. (2019). Absence of compulsive drinking phenotype in adult male rats exposed to ethanol in a binge-like pattern during adolescence. Alcohol (Fayetteville N.Y.).

[96] Cocker P.J., Hosking J.G., Murch W.S., Clark L., Winstanley C.A. (2016). Activation of dopamine D4 receptors within the anterior cingulate cortex enhances the erroneous expectation of reward on a rat slot machine task. Neuropharmacology.

[97] Cocker P.J., Lin M.Y., Barrus M.M., Le Foll B., Winstanley C.A. (2016). The agranular and granular insula differentially contribute to gambling-like behavior on a rat slot machine task: Effects of inactivation and local infusion of a dopamine D4 agonist on reward expectancy. Psychopharmacology.

[98] Rafa D., Kregiel J., Popik P., Rygula R. (2016). Effects of optimism on gambling in the rat slot machine task. Behav. Brain Res..

[99] Islas-Preciado D., Wainwright S.R., Sniegocki J., Lieblich S.E., Yagi S., Floresco S.B., Galea L.A.M. (2020). Risk-based decision making in rats: Modulation by sex and amphetamine. Horm. Behav..

[100] Orsini C.A., Willis M.L., Gilbert R.J., Bizon J.L., Setlow B. (2016). Sex differences in a rat model of risky decision making. Behav. Neurosci..

[101] Cardinal R.N., Howes N.J. (2005). Effects of lesions of the nucleus accumbens core on choice between small certain rewards and large uncertain rewards in rats. BMC Neurosci..

[102] Leenaars C.H., Dematteis M., Joosten R.N., Eggels L., Sandberg H., Schirris M., Feenstra M.G., Van Someren E.J. (2011). A new automated method for rat sleep deprivation with minimal confounding effects on corticosterone and locomotor activity. J. Neurosci. Methods.

[103] R_Core_Team (2020). R: A Language and Environment for Statistical Computing.

[104] Wickham H., Bryan J. readxl: Read Excel Files. R Package Version 1.3.1. https://CRAN.R-project.org/package=readxl.

[105] Wickham H., François R., Henry L., Müller K. dplyr: A Grammar of Data Manipulation. R Package Version 0.8.5. https://CRAN.R-project.org/package=dplyr.

[106] Wickham H. (2016). ggplot2: Elegant Graphics for Data Analysis.

