# Supplementary material for: Risk-Based Decision Making: A Systematic Scoping Review of Animal Models and a Pilot Study on the Effects of Sleep Deprivation in Rats
Source: Clocks Sleep. 2021 Jan 20;3(1):31–52. doi: 10.3390/clockssleep3010003 (PMC7838799; doi:10.3390/clockssleep3010003)
Supplement: Supplementary file 1 [file clockssleep-03-00003-s001.pdf]

Supplement: lists of included papers

1. Publications on gambling tasks in NHPs
2. Publications on gambling tasks in rodents
3. Publications on gambling tasks in birds
4. Publications on gambling tasks in other species

1: Publications on gambling tasks in NHPs

authors	year	title	journal	volume	issue	pages	Species	Sex
Azab, H. and Hayden, B. Y.	2018	Correlates of economic decisions in the dorsal and subgenual anterior cingulate cortices	Eur J Neurosci	47	8	979-993	Rhesus Macaques	na
Beran, Michael J. and Perdue, Bonnie M. and Parrish, Audrey E. and Evans, Theodore A.	2012	Do social conditions affect capuchin monkeys' (Cebus apella) choices in a quantity judgment task?	Frontiers in Psychology Vol 3 2012, ArtID 492	3		1-7	Capuchin Monkey	Both
Blanchard, Tommy C. and Wilke, Andreas and Hayden, Benjamin Y.	2014	Hot-hand bias in rhesus monkeys	Journal of Experimental Psychology: Animal Learning and Cognition	40	3	280-286	Rhesus Monkey	Male
Broihanne, M. H. and Romain, A. and Call, J. and Thierry, B. and Wascher, C. A. F. and De Marco, A. and Verrier, D. and Dufour, V.	2019	Monkeys (Sapajus apella and Macaca tonkeana) and great apes (Gorilla gorilla, Pongo abelii, Pan paniscus, and Pan troglodytes) play for the highest bid	J Comp Psychol	133	3	301-312	Several species	Both
Chen, X. and Stuphorn, V.	2018	Inactivation of Medial Frontal Cortex Changes Risk Preference	Curr Biol	28	19	3114-3122.e4	Monkey (Macaca mulatta)	na
De Petrillo, F. and Ventricelli, M. and Ponsi, G. and Addessi, E.	2015	Do tufted capuchin monkeys play the odds? Flexible risk preferences in Sapajus spp	Anim Cogn	18	1	119-30	Capuchin Monkey	Both
Farashahi, S. and Azab, H. and Hayden, B. and Soltani, A.	2018	On the Flexibility of Basic Risk Attitudes in Monkeys	J Neurosci	38	18	4383-4398	Monkey	Male
Farashahi, S. and Azab, H. and Hayden, B. and Soltani, A.	2018	On the Flexibility of Basic Risk Attitudes in Monkeys	J Neurosci	38	18	4383-4398	Monkey	Male
Ferrari-Toniolo, S. and Bujold, P. M. and Schultz, W.	2019	Probability Distortion Depends on Choice Sequence in Rhesus Monkeys	J Neurosci	39	15	2915-2929	Rhesus macaques	Both
Fiorillo, C. D.	2011	Transient activation of midbrain dopamine neurons by reward risk	Neuroscience	197		162-71	rhesus macaque	Both
Genest, W. and Stauffer, W. R. and Schultz, W.	2016	Utility functions predict variance and skewness risk preferences in monkeys	Proc Natl Acad Sci U S A	113	30	8402-7	Rhesus monkey	Male
Grabenhorst, F. and Tsutsui, K. I. and Kobayashi,	2019	Primate prefrontal neurons signal economic risk	Elife	8		1-30	Macaque	Male

authors	year	title	journal	volume	issue	pages	Species	Sex
S. and Schultz, W.		derived from the statistics of recent reward experience					Monkey	
Haun, D. B. and Nawroth, C. and Call, J.	2011	Great apes' risk-taking strategies in a decision making task	PLoS One	6	12	e28801	Chimpanzee, Bonobo, Orangutan, Gorilla	Both
Hayden, Benjamin Y. and Heilbronner, Sarah R. and Nair, Amrita C. and Platt, Michael L.	2008	Cognitive influences on risk-seeking by rhesus macaques	Judgment and Decision Making	3	5	389-395	Rhesus macaques	Male
Heilbronner, S. R. and Hayden, B. Y.	2016	The description-experience gap in risky choice in nonhuman primates	Psychon Bull Rev	23	2	593-600	Rhesus macaques	Male
Heilbronner, S. R. and Rosati, A. G. and Stevens, J. R. and Hare, B. and Hauser, M. D.	2008	A fruit in the hand or two in the bush? Divergent risk preferences in chimpanzees and bonobos	Biol Lett	4	3	246-9	Chimpanzee, Bonobo	Both
Kim, S. and Bobeica, I. and Gamo, N. J. and Arnsten, A. F. and Lee, D.	2012	Effects of alpha-2A adrenergic receptor agonist on time and risk preference in primates	Psychopharmacology (Berl)	219	2	363-75	Rhesus macaques	Male
Lakshminarayanan, Venkat R. and Chen, M. and Santos, Laurie R.	2011	The evolution of decision-making under risk: Framing effects in monkey risk preferences	Journal of Experimental Social Psychology	47	3	689-693	Capuchin Monkey	na
Long, A. B. and Kuhn, C. M. and Platt, M. L.	2009	Serotonin shapes risky decision making in monkeys	Soc Cogn Affect Neurosci	4	4	346-56	Rhesus macaques	Male
MacLean, E. L. and Mandalaywala, T. M. and Brannon, E. M.	2012	Variance-sensitive choice in lemurs: constancy trumps quantity	Anim Cogn	15	1	15-25	Lemur	Male
O'Neill, M. and Schultz, W.	2010	Coding of reward risk by orbitofrontal neurons is mostly distinct from coding of reward value	Neuron	68	4	789-800	Rhesus monkey	Male
Pele, M. and Broihanne, M. and Thierry, B. and Call, J. and Dufour, V.	2014	To bet or not to bet? Decision-making under risk in non-human primates	Journal of Risk and Uncertainty	49	2	141-166	Capuchin Monkey, Macaques, Orang-utans	Both
Perdue, B. M. and Brown, E. R.	2018	Irrational choice behavior in human and nonhuman primates	Anim Cogn	21	2	227-234	Capuchins, Rhesus macaques	Both
Proctor, Darby	2013	Gambling and decision-making among primates: The primate gambling task	Dissertation Abstracts International: Section B: The Sciences and Engineering	74	1	No Pagination Specified	Chimpanzees, Capuchin Monkey	Both
Raghuraman, A. P. and Padoa-Schioppa, C.	2014	Integration of multiple determinants in the neuronal computation of economic values	J Neurosci	34	35	11583-603	Rhesus macaques	Both
Riviere, J. and Stomp, M. and Augustin, E. and Lemasson, A. and Blois-Heulin, C.	2018	Decision-making under risk of gain in young children and mangabey monkeys	Dev Psychobiol	60	2	176-186	red-capped mangabeys	Both

authors	year	title	journal	volume	issue	pages	Species	Sex
Rosati, Alexandra G. and Hare, Brian	2012	Decision making across social contexts: Competition increases preferences for risk in chimpanzees and bonobos	Animal Behaviour	84	4	869-879	chimpanzees, bonobos	Both
Rosati, A. G. and Hare, B.	2013	Chimpanzees and bonobos exhibit emotional responses to decision outcomes	PLoS One	8	5	e63058	Chimpanzee, Bonobo	Both
Smith, T. R. and Beran, M. J.	2020	Outcome expectancy and suboptimal risky choice in nonhuman primates	Learn Behav	pre	pre	pre	rhesus macaques, capucin monkeys	Both
Smith, T. R. and Beran, M. J. and Young, M. E.	2017	Gambling in rhesus macaques (Macaca mulatta): The effect of cues signaling risky choice outcomes	Learn Behav	45	3	288-299	rhesus macaques	Male
So, N. and Stuphorn, V.	2016	Supplementary Eye Field Encodes Confidence in Decisions Under Risk	Cereb Cortex	26	2	764-82	rhesus monkey	Male
Stauffer, W. R. and Lak, A. and Bossaerts, P. and Schultz, W.	2015	Economic choices reveal probability distortion in macaque monkeys	J Neurosci	35	7	3146-54	Macaque Monkey	Male
Stauffer, W. R. and Lak, A. and Schultz, W.	2014	Dopamine reward prediction error responses reflect marginal utility	Curr Biol	24	21	2491-500	rhesus monkey	Male
Strait, C. E. and Hayden, B. Y.	2013	Preference patterns for skewed gambles in rhesus monkeys	Biol Lett	9	6	1-4	Rhesus macaques	na
Tokuno, H. and Tanaka, I.	2011	Decision making and risk attitude of the common marmoset in a gambling task	Neurosci Res	71	3	260-5	Marmoset	Both
Xu, E. R. and Kralik, J. D.	2014	Risky business: rhesus monkeys exhibit persistent preferences for risky options	Front Psychol	5		258	Rhesus monkey	Male
Yamada, H. and Tymula, A. and Louie, K. and Glimcher, P. W.	2013	Thirst-dependent risk preferences in monkeys identify a primitive form of wealth	Proc Natl Acad Sci U S A	110	39	15788-93	Rhesus monkey	na
Yanike, M. and Ferrera, V. P.	2014	Representation of outcome risk and action in the anterior caudate nucleus	J Neurosci	34	9	3279-90	rhesus monkey	Male
Zoratto, F. and Oddi, G. and Gori, E. and Micucci, A. and De Petrillo, F. and Paglieri, F. and Adriani, W. and Laviola, G. and Addessi, E.	2018	Social modulation of risky decision-making in rats (Rattus norvegicus) and tufted capuchin monkeys (Sapajus spp.)	Behav Brain Res	347		37-48	tufted capuchin monkey	Both
Zoratto, F. and Sinclair, E. and Manciocco, A. and Vitale, A. and Laviola, G. and Adriani, W.	2014	Individual differences in gambling proneness among rats and common marmosets: an automated choice task	Biomed Res Int	2014		1-12	Marmoset	Both

2: Publications on gambling tasks in rodents

authors	year	title	journal	volume	issue	pages	Species	Sex

authors	year	title	journal	volume	issue	pages	Species	Sex
Adams, W. K. and Barkus, C. and Ferland, J. N. and Sharp, T. and Winstanley, C. A.	2017	Pharmacological evidence that 5-HT2C receptor blockade selectively improves decision making when rewards are paired with audiovisual cues in a rat gambling task	Psychopharmacology (Berl)	234	20	3091-3104	Rat	Male
Adams, W. K. and Vonder Haar, C. and Tremblay, M. and Cocker, P. J. and Silveira, M. M. and Kaur, S. and Baunez, C. and Winstanley, C. A.	2017	Deep-Brain Stimulation of the Subthalamic Nucleus Selectively Decreases Risky Choice in Risk-Preferring Rats	eNeuro	4	4	1-13	Rat	Male
Adriani, W. and Boyer, F. and Gioiosa, L. and Macri, S. and Dreyer, J. L. and Laviola, G.	2009	Increased impulsive behavior and risk proneness following lentivirus-mediated dopamine transporter over-expression in rats' nucleus accumbens	Neuroscience	159	1	47-58	Rat	Male
Ashenhurst, James Rowan	2014	Translational examination of risk-related decision-making as an endophenotype for alcohol use disorders	Dissertation Abstracts International: Section B: The Sciences and Engineering	75	4	1-194	Rat	Male
Ashenhurst, J. R. and Seaman, M. and Jentsch, J. D.	2012	Responding in a test of decision-making under risk is under moderate genetic control in the rat	Alcohol Clin Exp Res	36	6	941-9	Rat	Both
Baarendse, P. J. and Winstanley, C. A. and Vanderschuren, L. J.	2013	Simultaneous blockade of dopamine and noradrenaline reuptake promotes disadvantageous decision making in a rat gambling task	Psychopharmacology (Berl)	225	3	719-31	Rat	Male
Barrus, M. M. and Hosking, J. G. and Cocker, P. J. and Winstanley, C. A.	2017	Inactivation of the orbitofrontal cortex reduces irrational choice on a rodent Betting Task	Neuroscience	345		38-48	Rat	Male
Barrus, M. M. and Hosking, J. G. and Zeeb, F. D. and Tremblay, M. and Winstanley, C. A.	2015	Disadvantageous decision-making on a rodent gambling task is associated with increased motor impulsivity in a population of male rats	J Psychiatry Neurosci	40	2	108-17	Rat	Male
Barrus, M. M. and Winstanley, C. A.	2016	Dopamine D3 Receptors Modulate the Ability of Win-Paired Cues to Increase Risky Choice in a Rat Gambling Task	J Neurosci	36	3	785-94	Rat	Male
Bercovici, D. A. and Princz-Lebel, O. and Tse, M. T. and Moorman, D. E. and Floresco, S. B.	2018	Optogenetic Dissection of Temporal Dynamics of Amygdala-Striatal Interplay during Risk/Reward Decision Making	eNeuro	5	6	1-16	Rat	Male
Blaes, S. L. and Orsini, C. A. and Mitchell, M. R. and Spurrell, M. S. and Betzhold, S. M. and Vera, K. and Bizon, J. L. and Setlow, B.	2018	Monoaminergic modulation of decision-making under risk of punishment in a rat model	Behav Pharmacol	29	8	745-761	Rat	Male
Boutros, N. and Der-Avakian, A. and Semenova, S. and Lee, S. and Markou, A.	2016	Risky choice and brain CRF after adolescent ethanol vapor exposure and social stress in adulthood	Behav Brain Res	311		160-166	Rat	Male
Boutros, N. and Semenova, S. and Liu, W. and	2014	Adolescent intermittent ethanol exposure is	Int J	18	2	1-9	Rat	Male

authors	year	title	journal	volume	issue	pages	Species	Sex
Crews, F. T. and Markou, A.		associated with increased risky choice and decreased dopaminergic and cholinergic neuron markers in adult rats	Neuropsychopharmacol					
Braunscheidel, K. M. and Okas, M. P. and Hoffman, M. and Mulholland, P. J. and Floresco, S. B. and Woodward, J. J.	2019	The Abused Inhalant Toluene Impairs Medial Prefrontal Cortex Activity and Risk/Reward Decision-Making during a Probabilistic Discounting Task	J Neurosci	39	46	9207-9220	Rat	Both
Breysse, E. and Meffre, J. and Pelloux, Y. and Winstanley, C. A. and Baunez, C.	2020	Decreased risk-taking and loss-chasing after subthalamic nucleus lesion in rats	Eur J Neurosci	0		e1-14	Rat	Male
Breysse, E. and Meffre, J. and Pelloux, Y. and Winstanley, C. A. and Baunez, C.	2020	Decreased risk-taking and loss-chasing after subthalamic nucleus lesion in rats	Eur J Neurosci	0		e1-14	Rat	Male
Cao, B. and Wang, J. and Shahed, M. and Jelfs, B. and Chan, R. H. and Li, Y.	2016	Vagus Nerve Stimulation Alters Phase Synchrony of the Anterior Cingulate Cortex and Facilitates Decision Making in Rats	Sci Rep	6		35135	Rat	Male
Cardinal, R. N. and Howes, N. J.	2005	Effects of lesions of the nucleus accumbens core on choice between small certain rewards and large uncertain rewards in rats	BMC Neurosci	6		37	Rat	Male
Cho, Bo Ram and Kwak, Myung Ji and Kim, Wha Young and Kim, Jeong-Hoon	2018	Impulsive action and impulsive choice are differentially expressed in rats depending on the age at exposure to a gambling task	Frontiers in Psychiatry Vol 9 2018, ArtID 503	9		1-11	Rat	Male
Clark, J. J. and Nasrallah, N. A. and Hart, A. S. and Collins, A. L. and Bernstein, I. L. and Phillips, P. E.	2012	Altered risk-based decision making following adolescent alcohol use results from an imbalance in reinforcement learning in rats	PLoS One	7	5	e37357	Rat	Male
Cocker, P. J. and Dinelle, K. and Kornelson, R. and Sossi, V. and Winstanley, C. A.	2012	Irrational choice under uncertainty correlates with lower striatal D(2/3) receptor binding in rats	J Neurosci	32	44	15450-7	Rat	Male
Constantinople, C. M. and Piet, A. T. and Bibawi, P. and Akrami, A. and Kopec, C. and Brody, C. D.	2019	Lateral orbitofrontal cortex promotes trial-by-trial learning of risky, but not spatial, biases	Elife	8		1-18	Rat	Male
Constantinople, C. M. and Piet, A. T. and Brody, C. D.	2019	An Analysis of Decision under Risk in Rats	Curr Biol	29	12	2066-2074.e5	Rat	Male
Cooper, S. E. and Goings, S. P. and Kim, J. Y. and Wood, R. I.	2014	Testosterone enhances risk tolerance without altering motor impulsivity in male rats	Psychoneuroendocrinology	40		201-12	Rat	Male
Corwin, Samantha Danielle	2019	Effect of adolescent alcohol use on encoding of decision-related variables in prefrontal cortex	Dissertation Abstracts International: Section B: The Sciences and Engineering	80	7	1-147	Rat	Both
Cui, R. and Wang, L. and Liu, L. and Ruan, H. and Li, X.	2018	Effects of noradrenergic and serotonergic systems on risk-based decision-making and center arena activity in open field in rats	Eur J Pharmacol	841		57-66	Rat	Male

authors	year	title	journal	volume	issue	pages	Species	Sex
Daniel, M. and Cocker, P. and Lacoste, J. and Mar, A. and Houeto, J. and Belin-Rauscent, A. and Belin, D.	2017	The anterior insula bidirectionally modulates cost-benefit decision-making on a rodent gambling task	European Journal of Neuroscience	46	10	2620-2628	Rat	Male
Dellu-Hagedorn, F. and Rivalan, M. and Fitoussi, A. and De Deurwaerdere, P.	2018	Inter-individual differences in the impulsive/compulsive dimension: deciphering related dopaminergic and serotonergic metabolisms at rest	Philos Trans R Soc Lond B Biol Sci	373	1744	1-9	Rat	na
Deng, J. V. and Orsini, C. A. and Shimp, K. G. and Setlow, B.	2018	MeCP2 Expression in a Rat Model of Risky Decision Making	Neuroscience	369		212-221	Rat	Male
Di Ciano, P. and Cormick, P. M. and Stefan, C. and Wong, E. and Kim, A. and Remington, G. and Le Foll, B.	2017	The effects of buspirone on occupancy of dopamine receptors and the rat gambling task	Psychopharmacology (Berl)	234	22	3309-3320	Rat	Male
Di Ciano, P. and Le Foll, B.	2016	Evaluating the Impact of Naltrexone on the Rat Gambling Task to Test Its Predictive Validity for Gambling Disorder	PLoS One	11	5	e0155604	Rat	Male
Di Ciano, P. and Manvich, D. F. and Pushparaj, A. and Gappasov, A. and Hess, E. J. and Weinshenker, D. and Le Foll, B.	2018	Effects of disulfiram on choice behavior in a rodent gambling task: association with catecholamine levels	Psychopharmacology (Berl)	235	1	23-35	Rat	Male
Di Ciano, P. and Pushparaj, A. and Kim, A. and Hatch, J. and Masood, T. and Ramzi, A. and Khaled, M. A. and Boileau, I. and Winstanley, C. A. and Le Foll, B.	2015	The Impact of Selective Dopamine D2, D3 and D4 Ligands on the Rat Gambling Task	PLoS One	10	9	e0136267	Rat	Male
Drozd, R. and Cieslak, P. E. and Rychlik, M. and Rodriguez Parkitna, J. and Rygula, R.	2016	Cognitive Judgment Bias Interacts with Risk Based Decision Making and Sensitivity to Dopaminergic Challenge in Male Rats	Front Behav Neurosci	10		163	Rat	Male
Ferland, Jacqueline-Marie N. and Adams, Wendy K. and Murch, Spencer and Wei, Linda and Clark, Luke and Winstanley, Catharine A.	2018	Investigating the influence of 'losses disguised as wins' on decision making and motivation in rats	Behavioural Pharmacology	29	8	732-744	Rat	Male
Ferland, Jacqueline-Marie N. and Carr, Madison R. and Lee, Angela M. and Hoogeland, Myrthe E. and Winstanley, Catharine A. and Pattij, Tommy	2018	Examination of the effects of cannabinoid ligands on decision making in a rat gambling task	Pharmacology, Biochemistry and Behavior	170		87-97	Rat	Male
Ferland, J. N. and Hynes, T. J. and Hounjet, C. D. and Lindenbach, D. and Vonder Haar, C. and Adams, W. K. and Phillips, A. G. and Winstanley, C. A.	2019	Prior Exposure to Salient Win-Paired Cues in a Rat Gambling Task Increases Sensitivity to Cocaine Self-Administration and Suppresses Dopamine Efflux in Nucleus Accumbens: Support for the Reward Deficiency Hypothesis of Addiction	J Neurosci	39	10	1842-1854	Rat	Male
Fitoussi, A. and Renault, P. and Le Moine, C. and Coutureau, E. and Cador, M. and Dellu-Hagedorn,	2018	Inter-individual differences in decision-making, flexible and goal-directed behaviors: novel	Brain Struct Funct	223	2	897-912	Rat	Male

authors	year	title	journal	volume	issue	pages	Species	Sex
F.		insights within the prefronto-striatal networks						
Floresco, S. B. and Montes, D. R. and Tse, M. M. T. and van Holstein, M.	2018	Differential Contributions of Nucleus Accumbens Subregions to Cue-Guided Risk/Reward Decision Making and Implementation of Conditional Rules	J Neurosci	38	8	1901-1914	Rat	Male
Freels, Timothy G. and Gabriel, Daniel B. and Lester, Deranda B. and Simon, Nicholas W.	2020	Risky decision-making predicts dopamine release dynamics in nucleus accumbens shell	Neuropsychopharmacology	45	2	266-275	Rat	Male
Gabriel, D. B. K. and Freels, T. G. and Setlow, B. and Simon, N. W.	2019	Risky decision-making is associated with impulsive action and sensitivity to first-time nicotine exposure	Behav Brain Res	359		579-588	Rat	Male
Gueye, A. B. and Trigo, J. M. and Vemuri, K. V. and Makriyannis, A. and Le Foll, B.	2016	Effects of various cannabinoid ligands on choice behaviour in a rat model of gambling	Behav Pharmacol	27	2	258-69	Rat	Male
Hastjarjo, T. and Silberberg, A. and Hursh, S. R.	1990	Risky choice as a function of amount and variance in food supply	J Exp Anal Behav	53	1	155-61	Rat	Male
Holtz, N. A. and Tedford, S. E. and Persons, A. L. and Grasso, S. A. and Napier, T. C.	2016	Pharmacologically distinct pramipexole-mediated akinesia vs. risk-taking in a rat model of Parkinson's disease	Prog Neuropsychopharmacol Biol Psychiatry	70		77-84	Rat	Male
Homberg, J. R. and van den Bos, R. and den Heijer, E. and Suer, R. and Cuppen, E.	2008	Serotonin transporter dosage modulates long-term decision-making in rat and human	Neuropharmacology	55	1	80-4	Rat	Female
Hong, D. D. and Huang, W. Q. and Ji, A. A. and Yang, S. S. and Xu, H. and Sun, K. Y. and Cao, A. and Gao, W. J. and Zhou, N. and Yu, P.	2019	Neurons in rat orbitofrontal cortex and medial prefrontal cortex exhibit distinct responses in reward and strategy-update in a risk-based decision-making task	Metab Brain Dis	34	2	417-429	Rat	Male
Hynes, Tristan J. and Ferland, Jacqueline-Marie M. and Feng, Tanya L. and Adams, Wendy K. and Silveira, Mason M. and Tremblay, Melanie and Chernoff, Chloe S. and Brodie, Hannah G. and Ebsary, Sophie A. and Russell, Brittney and Kaur, Sukhbir and Winstanley, Catharine A.	2020	Chemogenetic inhibition of dopaminergic projections to the nucleus accumbens has sexually dimorphic effects in the rat gambling task	Behavioral Neuroscience	134	4	309-322	Rat	Both
Ishii, H. and Ohara, S. and Tobler, P. N. and Tsutsui, K. and Iijima, T.	2012	Inactivating anterior insular cortex reduces risk taking	J Neurosci	32	45	16031-9	Rat	Male
Ishii, H. and Ohara, S. and Tobler, P. N. and Tsutsui, K. and Iijima, T.	2015	Dopaminergic and serotonergic modulation of anterior insular and orbitofrontal cortex function in risky decision making	Neurosci Res	92		53-61	Rat	Male
Ishii, H. and Onodera, M. and Ohara, S. and Tsutsui, K. I. and Iijima, T.	2018	Sex Differences in Risk Preference and c-Fos Expression in Paraventricular Thalamic Nucleus of Rats During Gambling Task	Front Behav Neurosci	12		68	Rat	Both
Islas-Preciado, D. and Wainwright, S. R. and Sniegocki, J. and Lieblich, S. E. and Yagi, S. and	2020	Risk-based decision making in rats: Modulation by sex and amphetamine	Horm Behav	125		1E+05	Rat	Both

authors	year	title	journal	volume	issue	pages	Species	Sex
Floresco, S. B. and Galea, L. A. M.								
Ito, M. and Takatsuru, S. and Saeki, D.	2000	Choice between constant and variable alternatives by rats: effects of different reinforcer amounts and energy budgets	J Exp Anal Behav	73	1	79-92	Rat	Male
Jacobs-Brichford, E. and Manson, K. F. and Roitman, J. D.	2019	Effects of chronic cannabinoid exposure during adolescence on reward preference and mPFC activation in adulthood	Physiol Behav	199		395-404	Rat	Both
Jentsch, J. D. and Woods, J. A. and Groman, S. M. and Seu, E.	2010	Behavioral characteristics and neural mechanisms mediating performance in a rodent version of the Balloon Analog Risk Task	Neuropsychopharmacology	35	8	1797-806	Rat	Male
Johnson, P. S. and Madden, G. J. and Brewer, A. T. and Pinkston, J. W. and Fowler, S. C.	2011	Effects of acute pramipexole on preference for gambling-like schedules of reinforcement in rats	Psychopharmacology (Berl)	213	1	44054	Rat	Male
Kagel, John H. and MacDonald, Don N. and Battalio, Raymond C. and White, Steven and Green, Leonard	1986	Risk aversion in rats (Rattus norvegicus) under varying levels of resource availability	Journal of Comparative Psychology	100	2	95-100	Rat	Male
Kaminski, B. J. and Ator, N. A.	2001	Behavioral and pharmacological variables affecting risky choice in rats	J Exp Anal Behav	75	3	275-97	Rat	Male
Kim, W. Y. and Cho, B. R. and Kwak, M. J. and Kim, J. H.	2017	Interaction between trait and housing condition produces differential decision-making toward risk choice in a rat gambling task	Sci Rep	7	1	5718	Rat	Male
Kirkpatrick, K. and Marshall, A. T. and Smith, A. P. and Koci, J. and Park, Y.	2014	Individual differences in impulsive and risky choice: effects of environmental rearing conditions	Behav Brain Res	269		115-27	Rat	Male
Koot, S. and Baars, A. and Hesseling, P. and van den Bos, R. and Joels, M.	2013	Time-dependent effects of corticosterone on reward-based decision-making in a rodent model of the Iowa Gambling Task	Neuropharmacology	70		306-15	Rat	Male
Koot, S. and Zoratto, F. and Cassano, T. and Colangeli, R. and Laviola, G. and van den Bos, R. and Adriani, W.	2012	Compromised decision-making and increased gambling proneness following dietary serotonin depletion in rats	Neuropharmacology	62	4	1640-50	Rat	Male
Koot, S. and Zoratto, F. and Cassano, T. and Colangeli, R. and Laviola, G. and van den Bos, R. and Adriani, W.	2012	Compromised decision-making and increased gambling proneness following dietary serotonin depletion in rats	Neuropharmacology	62	4	1640-50	Rat	Male
Langdon, Angela J. and Hathaway, Brett A. and Zorowitz, Samuel and Harris, Cailean B. and Winstanley, Catharine A.	2019	Relative insensitivity to time-out punishments induced by win-paired cues in a rat gambling task	Psychopharmacology	236	8	2543-2556	Rat	Male
Larkin, J. D. and Jenni, N. L. and Floresco, S. B.	2016	Modulation of risk/reward decision making by dopaminergic transmission within the basolateral amygdala	Psychopharmacology (Berl)	233	1	121-36	Rat	Male
Leblond, M. and Fan, D. and Brynildsen, J. K. and	2011	Motivational state and reward content determine	PLoS One	6	9	e25342	Mouse	Male

authors	year	title	journal	volume	issue	pages	Species	Sex
Yin, H. H.		choice behavior under risk in mice						
Leblond, M. and Sukharnikova, T. and Yu, C. and Rossi, M. A. and Yin, H. H.	2014	The role of pedunculopontine nucleus in choice behavior under risk	Eur J Neurosci	39	10	1664-70	Rat	na
Loi, M. and Mossink, J. C. and Meerhoff, G. F. and Den Blaauwen, J. L. and Lucassen, P. J. and Joels, M.	2017	Effects of early-life stress on cognitive function and hippocampal structure in female rodents	Neuroscience	342		101-119	Rat	Female
Mai, B. and Hauber, W.	2012	Intact risk-based decision making in rats with prefrontal or accumbens dopamine depletion	Cogn Affect Behav Neurosci	12	4	719-29	Rat	Male
Mai, B. and Hauber, W.	2015	Orbitofrontal or accumbens dopamine depletion does not affect risk-based decision making in rats	Cogn Affect Behav Neurosci	15	3	507-22	Rat	Male
Mai, Bettina and Sommer, Susanne and Hauber, Wolfgang	2015	Dopamine D1/D2 receptor activity in the nucleus accumbens core but not in the nucleus accumbens shell and orbitofrontal cortex modulates risk-based decision making	International Journal of Neuropsychopharmacology	18	10	44075	Rat	Male
Marshall, Andrew T. and Kirkpatrick, Kimberly	2013	The effects of the previous outcome on probabilistic choice in rats	Journal of Experimental Psychology: Animal Behavior Processes	39	1	24-38	Rat	Male
Marshall, A. T. and Kirkpatrick, K.	2017	Reinforcement learning models of risky choice and the promotion of risk-taking by losses disguised as wins in rats	J Exp Psychol Anim Learn Cogn	43	3	262-279	Rat	Male
Mazur, James E.	1988	Choice between small certain and large uncertain reinforcers	Animal Learning & Behavior	16	2	199-205	Rat	Male
McMurray, M. S. and Amodeo, L. R. and Roitman, J. D.	2014	Effects of voluntary alcohol intake on risk preference and behavioral flexibility during rat adolescence	PLoS One	9	7	e100697	Rat	Male
McMurray, M. S. and Amodeo, L. R. and Roitman, J. D.	2016	Consequences of Adolescent Ethanol Consumption on Risk Preference and Orbitofrontal Cortex Encoding of Reward	Neuropsychopharmacology	41	5	1366-75	Rat	Male
Mendez, Ian Alfredo	2011	The roles of nicotinic and muscarinic cholinergic receptors in risky and impulsive decision making	Dissertation Abstracts International: Section B: The Sciences and Engineering	72	5	2601	Rat	Male
Milienne-Petiot, M. and Geyer, M. A. and Arnt, J. and Young, J. W.	2017	Brexpiprazole reduces hyperactivity, impulsivity, and risk-preference behavior in mice with dopamine transporter knockdown-a model of mania	Psychopharmacology (Berl)	234	6	1017-1028	Mouse	Both
Miller, K. M. and Risher, M. L. and Acheson, S. K.	2017	Behavioral Inefficiency on a Risky Decision-	Sci Rep	7	1	4680	Rat	Male

authors	year	title	journal	volume	issue	pages	Species	Sex
and Darlow, M. and Sexton, H. G. and Schramm-Sapyta, N. and Swartzwelder, H. S.		Making Task in Adulthood after Adolescent Intermittent Ethanol Exposure in Rats						
Mitchell, M. R. and Vokes, C. M. and Blankenship, A. L. and Simon, N. W. and Setlow, B.	2011	Effects of acute administration of nicotine, amphetamine, diazepam, morphine, and ethanol on risky decision-making in rats	Psychopharmacology (Berl)	218	4	703-12	Rat	Male
Mitchell, M. R. and Weiss, V. G. and Beas, B. S. and Morgan, D. and Bizon, J. L. and Setlow, B.	2014	Adolescent risk taking, cocaine self-administration, and striatal dopamine signaling	Neuropsychopharmacology	39	4	955-62	Rat	Male
Mizoguchi, H. and Katahira, K. and Inutsuka, A. and Fukumoto, K. and Nakamura, A. and Wang, T. and Nagai, T. and Sato, J. and Sawada, M. and Ohira, H. and Yamanaka, A. and Yamada, K.	2015	Insular neural system controls decision-making in healthy and methamphetamine-treated rats	Proc Natl Acad Sci U S A	112	29	E3930-9	Rat	Male
Mizoguchi, H. and Wang, T. and Kusaba, M. and Fukumoto, K. and Yamada, K.	2019	Nicotine and varenicline ameliorate changes in reward-based choice strategy and altered decision-making in methamphetamine-treated rats	Behav Brain Res	359		935-941	Rat	Male
Montes, D. R. and Stopper, C. M. and Floresco, S. B.	2015	Noradrenergic modulation of risk/reward decision making	Psychopharmacology (Berl)	232	15	2681-96	Rat	Male
Morgado, P. and Marques, F. and Ribeiro, B. and Leite-Almeida, H. and Pego, J. M. and Rodrigues, A. J. and Dalla, C. and Kokras, N. and Sousa, N. and Cerqueira, J. J.	2015	Stress induced risk-aversion is reverted by D2/D3 agonist in the rat	Eur Neuropsychopharmacol	25	10	1744-52	Rat	Male
Morgado, P. and Marques, F. and Silva, M. B. and Sousa, N. and Cerqueira, J. J.	2014	A novel risk-based decision-making paradigm	Front Behav Neurosci	8		45	Rat	Male
Mu, L. and Wang, J. and Cao, B. and Jelfs, B. and Chan, R. H. and Xu, X. and Hasan, M. and Zhang, X. and Li, Y.	2015	Impairment of cognitive function by chemotherapy: association with the disruption of phase-locking and synchronization in anterior cingulate cortex	Mol Brain	8		32	Rat	Male
Nasrallah, N. A. and Clark, J. J. and Collins, A. L. and Akers, C. A. and Phillips, P. E. and Bernstein, I. L.	2011	Risk preference following adolescent alcohol use is associated with corrupted encoding of costs but not rewards by mesolimbic dopamine	Proc Natl Acad Sci U S A	108	13	5466-71	Rat	Male
Nasrallah, N. A. and Yang, T. W. and Bernstein, I. L.	2009	Long-term risk preference and suboptimal decision making following adolescent alcohol use	Proc Natl Acad Sci U S A	106	41	17600-4	Rat	Male
Nobrega, J. N. and Hedayatmofidi, P. S. and Lobo, D. S.	2016	Strong interactions between learned helplessness and risky decision-making in a rat gambling model	Sci Rep	6		37304	Rat	Male
Oggiano, Maurizio and Zoratto, Francesca and Palombelli, Gianmauro and Festucci, Fabiana and Laviola, Giovanni and Curcio, Giuseppe and	2020	Striatal dynamics as determinants of reduced gambling vulnerability in the NHE rat model of ADHD	Progress in Neuro-Psychopharmacology & Biological	100		1-9	Rat	Male

authors	year	title	journal	volume	issue	pages	Species	Sex
Canese, Rossella and Adriani, Walter			Psychiatry Vol 100 2020, ArtID 109886					
Olshavsky, M. E. and Shumake, J. and Rosenthal, A. A. and Kaddour-Djebbar, A. and Gonzalez-Lima, F. and Setlow, B. and Lee, H. J.	2014	Impulsivity, risk-taking, and distractibility in rats exhibiting robust conditioned orienting behaviors	J Exp Anal Behav	102	2	162-78	Rat	Male
Orduna, V. and Alba, R.	2019	Rats' optimal choice behavior in a gambling-like task	Behav Processes	162		104-111	Rat	Male
Orsini, C. A. and Blaes, S. L. and Dragone, R. J. and Betzhold, S. M. and Finner, A. M. and Bizon, J. L. and Setlow, B.	2020	Distinct relationships between risky decision making and cocaine self-administration under short- and long-access conditions	Prog Neuropsychopharmacol Biol Psychiatry	98		1E+05	Rat	Both
Orsini, C. A. and Blaes, S. L. and Setlow, B. and Simon, N. W.	2019	Recent Updates in Modeling Risky Decision Making in Rodents	Methods Mol Biol	2011		79-92	Rat	Both
Orsini, C. A. and Hernandez, C. M. and Singhal, S. and Kelly, K. B. and Frazier, C. J. and Bizon, J. L. and Setlow, B.	2017	Optogenetic Inhibition Reveals Distinct Roles for Basolateral Amygdala Activity at Discrete Time Points during Risky Decision Making	J Neurosci	37	48	11537-11548	Rat	Male
Orsini, C. A. and Heshmati, S. C. and Garman, T. S. and Wall, S. C. and Bizon, J. L. and Setlow, B.	2018	Contributions of medial prefrontal cortex to decision making involving risk of punishment	Neuropharmacology	139		205-216	Rat	Male
Orsini, C. A. and Trotta, R. T. and Bizon, J. L. and Setlow, B.	2015	Dissociable roles for the basolateral amygdala and orbitofrontal cortex in decision-making under risk of punishment	J Neurosci	35	4	1368-79	Rat	Male
Orsini, C. A. and Willis, M. L. and Gilbert, R. J. and Bizon, J. L. and Setlow, B.	2016	Sex differences in a rat model of risky decision making	Behav Neurosci	130	1	50-61	Rat	Both
Ozga-Hess, J. E. and Anderson, K. G.	2019	Differential effects of d-amphetamine and atomoxetine on risk-based decision making of Lewis and Fischer 344 rats	Behav Pharmacol	30	7	605-616	Rat	Male
Ozga-Hess, J. E. and Whirtley, C. and O'Hearn, C. and Pechacek, K. and Vonder Haar, C.	2020	Unilateral parietal brain injury increases risk-taking on a rat gambling task	Exp Neurol	327		1E+05	Rat	Male
Paine, T. and O'Hara, A. and Plaut, B. and Lowes, D.	2015	Effects of disrupting medial prefrontal cortex GABA transmission on decision-making in a rodent gambling task	Psychopharmacology	232	10	1755-1765	Rat	Male
Paine, T. A. and Asinof, S. K. and Diehl, G. W. and Frackman, A. and Leffler, J.	2013	Medial prefrontal cortex lesions impair decision-making on a rodent gambling task: reversal by D1 receptor antagonist administration	Behav Brain Res	243		247-54	Rat	Male
Pais-Vieira, M. and Aguiar, P. and Lima, D. and Galhardo, V.	2012	Inflammatory pain disrupts the orbitofrontal neuronal activity and risk-assessment performance in a rodent decision-making task	Pain	153	8	1625-35	Rat	Male
Pais-Vieira, M. and Mendes-Pinto, M. M. and Lima, D. and Galhardo, V.	2009	Cognitive impairment of prefrontal-dependent decision-making in rats after the onset of chronic pain	Neuroscience	161	3	671-9	Rat	Male

authors	year	title	journal	volume	issue	pages	Species	Sex
Parker, J. G. and Wanat, M. J. and Soden, M. E. and Ahmad, K. and Zweifel, L. S. and Bamford, N. S. and Palmiter, R. D.	2011	Attenuating GABA(A) receptor signaling in dopamine neurons selectively enhances reward learning and alters risk preference in mice	J Neurosci	31	47	17103-12	Mouse	Both
Passecker, J. and Mikus, N. and Malagon-Vina, H. and Anner, P. and Dimidschstein, J. and Fishell, G. and Dorffner, G. and Klausberger, T.	2019	Activity of Prefrontal Neurons Predict Future Choices during Gambling	Neuron	101	1	152-164.e7	Rat	Male
Peak, J. N. and Turner, K. M. and Burne, T. H.	2015	The effect of developmental vitamin D deficiency in male and female Sprague-Dawley rats on decision-making using a rodent gambling task	Physiol Behav	138		319-24	Rat	Both
Pena-Oliver, Y. and Sanchez-Roige, S. and Stephens, D. N. and Ripley, T. L.	2014	Alpha-synuclein deletion decreases motor impulsivity but does not affect risky decision making in a mouse Gambling Task	Psychopharmacology (Berl)	231	12	2493-506	Mouse	Male
Persons, A. L. and Tedford, S. E. and Celeste, T.	2017	Mirtazapine and ketanserin alter preference for gambling-like schedules of reinforcement in rats	Prog Neuropsychopharmacol Biol Psychiatry	77		178-184	Rat	Male
Pes, R. and Godar, S. C. and Fox, A. T. and Burgeno, L. M. and Strathman, H. J. and Jarmolowicz, D. P. and Devoto, P. and Levant, B. and Phillips, P. E. and Fowler, S. C. and Bortolato, M.	2017	Pramipexole enhances disadvantageous decision-making: Lack of relation to changes in phasic dopamine release	Neuropharmacology	114		77-87	Rat	Male
Piantadosi, P. T. and Khayambashi, S. and Schluter, M. G. and Kutarna, A. and Floresco, S. B.	2016	Perturbations in reward-related decision-making induced by reduced prefrontal cortical GABA transmission: Relevance for psychiatric disorders	Neuropharmacology	101		279-90	Rat	Male
Pittaras, E. and Callebert, J. and Chennaoui, M. and Rabat, A. and Granon, S.	2016	Individual behavioral and neurochemical markers of unadapted decision-making processes in healthy inbred mice	Brain Struct Funct	221	9	4615-4629	Mouse	Male
Pittaras, E. and Callebert, J. and Dorey, R. and Chennaoui, M. and Granon, S. and Rabat, A.	2018	Mouse Gambling Task reveals differential effects of acute sleep debt on decision-making and associated neurochemical changes	Sleep	41	11	1-14	Mouse	Male
Pittaras, E. C. and Faure, A. and Leray, X. and Moraitopoulou, E. and Cressant, A. and Rabat, A. A. and Meunier, C. and Fossier, P. and Granon, S.	2016	Neuronal Nicotinic Receptors Are Crucial for Tuning of E/I Balance in Prelimbic Cortex and for Decision-Making Processes	Front Psychiatry	7		171	Mouse	Male
Pushparaj, A. and Kim, A. S. and Musiol, M. and Zangen, A. and Daskalakis, Z. J. and Zack, M. and Winstanley, C. A. and Le Foll, B.	2015	Differential Involvement of the Agranular vs Granular Insular Cortex in the Acquisition and Performance of Choice Behavior in a Rodent Gambling Task	Neuropsychopharmacology	40	12	2832-42	Rat	Male
Ricker, J. M. and Hatch, J. D. and Powers, D. D.	2016	Fractionating choice: A study on reward	J Comp Psychol	130	2	174-86	Rat	Male

authors	year	title	journal	volume	issue	pages	Species	Sex
and Cromwell, H. C.		discrimination, preference, and relative valuation in the rat (Rattus norvegicus)						
Rivalan, M. and Ahmed, S. H. and Dellu-Hagedorn, F.	2009	Risk-prone individuals prefer the wrong options on a rat version of the Iowa Gambling Task	Biol Psychiatry	66	8	743-9	Rat	Male
Rivalan, M. and Valton, V. and Series, P. and Marchand, A. R. and Dellu-Hagedorn, F.	2013	Elucidating poor decision-making in a rat gambling task	PLoS One	8	12	e82052	Rat	Male
Roitman, J. D. and Roitman, M. F.	2010	Risk-preference differentiates orbitofrontal cortex responses to freely chosen reward outcomes	Eur J Neurosci	31	8	1492-500	Rat	Male
Rokosik, S. L. and Napier, T. C.	2011	Intracranial self-stimulation as a positive reinforcer to study impulsivity in a probability discounting paradigm	J Neurosci Methods	198	2	260-9	Rat	Male
Samson, R. D. and Venkatesh, A. and Lester, A. W. and Weinstein, A. T. and Lipa, P. and Barnes, C. A.	2015	Age differences in strategy selection and risk preference during risk-based decision making	Behav Neurosci	129	2	138-48	Rat	Male
Sanchez-Roige, Sandra and Pena-Oliver, Yolanda and Ripley, Tamzin L. and Stephens, David N.	2014	Repeated ethanol exposure during early and late adolescence: Double dissociation of effects on waiting and choice impulsivity	Alcoholism: Clinical and Experimental Research	38	10	2579-2589	Mouse	Male
Schindler, A. G. and Soden, M. E. and Zweifel, L. S. and Clark, J. J.	2016	Reversal of Alcohol-Induced Dysregulation in Dopamine Network Dynamics May Rescue Maladaptive Decision-making	J Neurosci	36	13	3698-708	Rat	Male
Schindler, A. G. and Tsutsui, K. T. and Clark, J. J.	2014	Chronic alcohol intake during adolescence, but not adulthood, promotes persistent deficits in risk-based decision making	Alcohol Clin Exp Res	38	6	1622-9	Rat	Male
Shaver, T. K. and Ozga, J. E. and Zhu, B. and Anderson, K. G. and Martens, K. M. and Vonder Haar, C.	2019	Long-term deficits in risky decision-making after traumatic brain injury on a rat analog of the Iowa gambling task	Brain Res	1704		103-113	Rat	Male
Shimp, Kristy G. and Mitchell, Marci R. and Beas, B. and Bizon, Jennifer L. and Setlow, Barry	2015	Affective and cognitive mechanisms of risky decision making	Neurobiology of Learning and Memory	117		60-70	Rat	Male
Silveira, M. M. and Malcolm, E. and Shoaib, M. and Winstanley, C. A.	2015	Scopolamine and amphetamine produce similar decision-making deficits on a rat gambling task via independent pathways	Behav Brain Res	281		86-95	Rat	Male
Silveira, M. M. and Murch, W. S. and Clark, L. and Winstanley, C. A.	2016	Chronic atomoxetine treatment during adolescence does not influence decision-making on a rodent gambling task, but does modulate amphetamine's effect on impulsive action in adulthood	Behav Pharmacol	27	4	350-63	Rat	Male
Simon, Nicholas Wayne	2010	D2 dopamine receptor mediation of risky decision-making	Dissertation Abstracts International: Section	71	8	1-119	Rat	Male

authors	year	title	journal	volume	issue	pages	Species	Sex
			B: The Sciences and Engineering					
Simon, N. W. and Gilbert, R. J. and Mayse, J. D. and Bizon, J. L. and Setlow, B.	2009	Balancing risk and reward: a rat model of risky decision making	Neuropsychopharmacology	34	10	2208-17	Rat	Male
Simon, N. W. and Montgomery, K. S. and Beas, B. S. and Mitchell, M. R. and LaSarge, C. L. and Mendez, I. A. and Banuelos, C. and Vokes, C. M. and Taylor, A. B. and Haberman, R. P. and Bizon, J. L. and Setlow, B.	2011	Dopaminergic modulation of risky decision-making	J Neurosci	31	48	17460-70	Rat	Male
Smith, A. P. and Hofford, R. S. and Zentall, T. R. and Beckmann, J. S.	2018	The role of 'jackpot' stimuli in maladaptive decision-making: dissociable effects of D1/D2 receptor agonists and antagonists	Psychopharmacology (Berl)	235	5	1427-1437	Rat	Male
Spoelder, M. and Flores Dourojeanni, J. P. and de Git, K. C. G. and Baars, A. M. and Lesscher, H. M. B. and Vanderschuren, Ljmj	2017	Individual differences in voluntary alcohol intake in rats: relationship with impulsivity, decision making and Pavlovian conditioned approach	Psychopharmacology (Berl)	234	14	2177-2196	Rat	Male
Spoelder, M. and Lesscher, H. M. and Hesseling, P. and Baars, A. M. and Lozeman-van t Klooster, J. G. and Mijnsbergen, R. and Vanderschuren, L. J.	2015	Altered performance in a rat gambling task after acute and repeated alcohol exposure	Psychopharmacology (Berl)	232	19	3649-62	Rat	Male
St Onge, J. R. and Abhari, H. and Floresco, S. B.	2011	Dissociable contributions by prefrontal D1 and D2 receptors to risk-based decision making	J Neurosci	31	23	8625-33	Rat	Male
St Onge, J. R. and Ahn, S. and Phillips, A. G. and Floresco, S. B.	2012	Dynamic fluctuations in dopamine efflux in the prefrontal cortex and nucleus accumbens during risk-based decision making	J Neurosci	32	47	16880-91	Rat	Male
St Onge, J. R. and Chiu, Y. C. and Floresco, S. B.	2010	Differential effects of dopaminergic manipulations on risky choice	Psychopharmacology (Berl)	211	2	209-21	Rat	Male
St Onge, J. R. and Floresco, S. B.	2009	Dopaminergic modulation of risk-based decision making	Neuropsychopharmacology	34	3	681-97	Rat	Male
St Onge, J. R. and Floresco, S. B.	2010	Prefrontal cortical contribution to risk-based decision making	Cereb Cortex	20	8	1816-28	Rat	Male
St Onge, J. R. and Stopper, C. M. and Zahm, D. S. and Floresco, S. B.	2012	Separate prefrontal-subcortical circuits mediate different components of risk-based decision making	J Neurosci	32	8	2886-99	Rat	Male
Stopper, C. M. and Floresco, S. B.	2011	Contributions of the nucleus accumbens and its subregions to different aspects of risk-based decision making	Cogn Affect Behav Neurosci	11	1	97-112	Rat	Male
Stopper, C. M. and Green, E. B. and Floresco, S. B.	2014	Selective involvement by the medial orbitofrontal cortex in biasing risky, but not impulsive, choice	Cereb Cortex	24	1	154-62	Rat	Male
Stopper, C. M. and Khayambashi, S. and	2013	Receptor-specific modulation of risk-based	Neuropsychopharmac	38	5	715-28	Rat	Male

authors	year	title	journal	volume	issue	pages	Species	Sex
Floresco, S. B.		decision making by nucleus accumbens dopamine	ology					
Stopper, C. M. and Tse, M. T. L. and Montes, D. R. and Wiedman, C. R. and Floresco, S. B.	2014	Overriding phasic dopamine signals redirects action selection during risk/reward decision making	Neuron	84	1	177-189	Rat	Male
Sugam, J. A. and Day, J. J. and Wightman, R. M. and Carelli, R. M.	2012	Phasic nucleus accumbens dopamine encodes risk-based decision-making behavior	Biol Psychiatry	71	3	199-205	Rat	Male
Sugam, J. A. and Saddoris, M. P. and Carelli, R. M.	2014	Nucleus accumbens neurons track behavioral preferences and reward outcomes during risky decision making	Biol Psychiatry	75	10	807-816	Rat	Male
Takahashi, K. and Toyoshima, M. and Ichitani, Y. and Yamada, K.	2020	Enhanced methamphetamine-induced conditioned place preference in risk-taking rats	Behav Brain Res	378		1E+05	Rat	Male
Tan, D. and Vyas, A.	2016	Toxoplasma gondii infection and testosterone congruently increase tolerance of male rats for risk of reward forfeiture	Horm Behav	79		37-44	Rat	Male
Tremblay, M. and Barrus, M. M. and Cocker, P. J. and Baunez, C. and Winstanley, C. A.	2019	Increased motor impulsivity in a rat gambling task during chronic ropinirole treatment: potentiation by win-paired audiovisual cues	Psychopharmacology (Berl)	236	6	1901-1915	Rat	Male
Tremblay, M. and Cocker, P. J. and Hosking, J. G. and Zeeb, F. D. and Rogers, R. D. and Winstanley, C. A.	2014	Dissociable effects of basolateral amygdala lesions on decision making biases in rats when loss or gain is emphasized	Cogn Affect Behav Neurosci	14	4	1184-95	Rat	na
Tremblay, M. and Cocker, P. J. and Hosking, J. G. and Zeeb, F. D. and Rogers, R. D. and Winstanley, C. A.	2014	Dissociable effects of basolateral amygdala lesions on decision making biases in rats when loss or gain is emphasized	Cogn Affect Behav Neurosci	14	4	1184-95	Rat	na
Tremblay, M. and Silveira, M. M. and Kaur, S. and Hosking, J. G. and Adams, W. K. and Baunez, C. and Winstanley, C. A.	2017	Chronic D2/3 agonist ropinirole treatment increases preference for uncertainty in rats regardless of baseline choice patterns	Eur J Neurosci	45	1	159-166	Rat	Male
Tremblay, M. and Winstanley, C. A.	2016	Anticonvulsant medications attenuate amphetamine-induced deficits in behavioral inhibition but not decision making under risk on a rat gambling task	Behav Brain Res	314		143-51	Rat	Male
Tryon, Valerie	2017	Investigating the contributions of hippocampal memory and reward valuation systems to cost-benefit decision making	Dissertation Abstracts International: Section B: The Sciences and Engineering	79	1	No Pagination Specified	Rat	Male
Tryon, Valerie	2017	Investigating the contributions of hippocampal memory and reward valuation systems to cost-benefit decision making	Dissertation Abstracts International: Section B: The Sciences and	79	1	No Pagination	Rat	Male

authors	year	title	journal	volume	issue	pages	Species	Sex
			Engineering			Specifi ed		
van den Bos, R. and Jolles, J. and van der Knaap, L. and Baars, A. and de Visser, L.	2012	Male and female Wistar rats differ in decision-making performance in a rodent version of the Iowa Gambling Task	Behav Brain Res	234	2	375-9	Rat	Both
van den Bos, R. and Lasthuis, W. and den Heijer, E. and van der Harst, J. and Spruijt, B.	2006	Toward a rodent model of the Iowa gambling task	Behav Res Methods	38	3	470-8	mouse, rat	Both
van Enkhuizen, J. and Geyer, M. A. and Young, J. W.	2013	Differential effects of dopamine transporter inhibitors in the rodent Iowa gambling task: relevance to mania	Psychopharmacology (Berl)	225	3	661-74	Mouse	Male
van Enkhuizen, J. and Henry, B. L. and Minassian, A. and Perry, W. and Milienne-Petiot, M. and Higa, K. K. and Geyer, M. A. and Young, J. W.	2014	Reduced dopamine transporter functioning induces high-reward risk-preference consistent with bipolar disorder	Neuropsychopharmac ology	39	13	3112-22	Mouse	Male
van Hasselt, F. N. and de Visser, L. and Tieskens, J. M. and Cornelisse, S. and Baars, A. M. and Lavrijsen, M. and Krugers, H. J. and van den Bos, R. and Joels, M.	2012	Individual variations in maternal care early in life correlate with later life decision-making and c-fos expression in prefrontal subregions of rats	PLoS One	7	5	e37820	Rat	Both
van Holstein, Mieke and Floresco, Stan B.	2020	Dissociable roles for the ventral and dorsal medial prefrontal cortex in cue-guided risk/reward decision making	Neuropsychopharmac ology	45	4	683-693	Rat	Male
Verharen, J. P. H. and de Jong, J. W. and Roelofs, T. J. M. and Huffels, C. F. M. and van Zessen, R. and Luijendijk, M. C. M. and Hamelink, R. and Willuhn, I. and den Ouden, H. E. M. and van der Plasse, G. and Adan, R. A. H. and Vanderschuren, Ljmj	2018	A neuronal mechanism underlying decision-making deficits during hyperdopaminergic states	Nat Commun	9	1	731	Rat	Male
Wallin, K. G. and Alves, J. M. and Wood, R. I.	2015	Anabolic-androgenic steroids and decision making: Probability and effort discounting in male rats	Psychoneuroendocrin ology	57		84-92	Rat	Male
Wallin-Miller, K. and Li, G. and Kelishani, D. and Wood, R. I.	2018	Anabolic-androgenic steroids alter decision making in a balanced rodent model of the Iowa gambling task	Behav Neurosci	132	3	152-160	Rat	Male
Wallin-Miller, K. G. and Chesley, J. and Castrillon, J. and Wood, R. I.	2017	Sex differences and hormonal modulation of ethanol-enhanced risk taking in rats	Drug Alcohol Depend	174		137-144	Rat	Both
Westbrook, S. R. and Hankosky, E. R. and Dwyer, M. R. and Gulley, J. M.	2018	Age and sex differences in behavioral flexibility, sensitivity to reward value, and risky decision-making	Behav Neurosci	132	2	75-87	Rat	Both
Yang, F. N. and Pan, J. S. and Li, X.	2016	Beta-adrenoreceptor blockade abolishes	Physiol Behav	153		125-32	Rat	Male

authors	year	title	journal	volume	issue	pages	Species	Sex
		atomoxetine-induced risk taking						
Yang, J. H. and Cheng, C. P. and Liao, R. M.	2018	Effects of d-amphetamine on risk choice in rats depend on the manner in which the expected reward value is varied	Pharmacol Biochem Behav	171		20-29	Rat	Male
Yang, J. H. and Liao, R. M.	2015	Dissociable contribution of nucleus accumbens and dorsolateral striatum to the acquisition of risk choice behavior in the rat	Neurobiol Learn Mem	126		67-77	Rat	Male
Yates, J. R. and Breitenstein, K. A. and Gunkel, B. T. and Hughes, M. N. and Johnson, A. B. and Rogers, K. K. and Shape, S. M.	2016	Effects of NMDA receptor antagonists on probability discounting depend on the order of probability presentation	Pharmacol Biochem Behav	150		31-38	Rat	Male
Yates, J. R. and Chitwood, M. R. and Evans, K. E. and Kappesser, J. L. and Murray, C. P. and Paradella-Bradley, T. A. and Torline, B. T.	2019	Group I metabotropic glutamate receptor antagonists impair discriminability of reinforcer magnitude, but not risky choice, in a probability-discounting task	Behav Brain Res	365		77-81	Rat	Male
Zalocusky, K. A. and Ramakrishnan, C. and Lerner, T. N. and Davidson, T. J. and Knutson, B. and Deisseroth, K.	2016	Nucleus accumbens D2R cells signal prior outcomes and control risky decision-making	Nature	531	7596	642-6	Rat	Male
Zeeb, F. D. and Baarendse, P. J. and Vanderschuren, L. J. and Winstanley, C. A.	2015	Inactivation of the prelimbic or infralimbic cortex impairs decision-making in the rat gambling task	Psychopharmacology (Berl)	232	24	4481-91	Rat	Male
Zeeb, F. D. and Li, Z. and Fisher, D. C. and Zack, M. H. and Fletcher, P. J.	2017	Uncertainty exposure causes behavioural sensitization and increases risky decision-making in male rats: toward modelling gambling disorder	J Psychiatry Neurosci	42	6	404-413	Rat	Male
Zeeb, F. D. and Winstanley, C. A.	2011	Lesions of the basolateral amygdala and orbitofrontal cortex differentially affect acquisition and performance of a rodent gambling task	J Neurosci	31	6	2197-204	Rat	Male
Zeeb, F. D. and Wong, A. C. and Winstanley, C. A.	2013	Differential effects of environmental enrichment, social-housing, and isolation-rearing on a rat gambling task: dissociations between impulsive action and risky decision-making	Psychopharmacology (Berl)	225	2	381-95	Rat	Male
Zoratto, F. and Laviola, G. and Adriani, W.	2012	Choice with delayed or uncertain reinforcers in rats: influence of timeout duration and session length	Synapse	66	9	792-806	Rat	Male
Zoratto, F. and Laviola, G. and Adriani, W.	2013	Gambling proneness in rats during the transition from adolescence to young adulthood: a home-cage method	Neuropharmacology	67		444-54	Rat	Male
Zoratto, F. and Laviola, G. and Adriani, W.	2016	The subjective value of probabilistic outcomes: Impact of reward magnitude on choice with	Neurosci Lett	617		225-31	Rat	Male

authors	year	title	journal	volume	issue	pages	Species	Sex
		uncertain rewards in rats						
Zoratto, F. and Oddi, G. and Gori, E. and Micucci, A. and De Petrillo, F. and Paglieri, F. and Adriani, W. and Laviola, G. and Addessi, E.	2018	Social modulation of risky decision-making in rats (Rattus norvegicus) and tufted capuchin monkeys (Sapajus spp.)	Behav Brain Res	347		37-48	Rat	Male
Zoratto, F. and Sinclair, E. and Manciocco, A. and Vitale, A. and Laviola, G. and Adriani, W.	2014	Individual differences in gambling proneness among rats and common marmosets: an automated choice task	Biomed Res Int	2014		1-12	Rat	Male

3: Publications on gambling tasks in birds

authors	year	title	journal	volume	issue	pages	Species	Sex
Aw, J. and Monteiro, T. and Vasconcelos, M. and Kacelnik, A.	2012	Cognitive mechanisms of risky choice: is there an evaluation cost?	Behav Processes	89	2	95-103	Starling	na
Gipson, C. D. and Alessandri, J. J. and Miller, H. C. and Zentall, T. R.	2009	Preference for 50% reinforcement over 75% reinforcement by pigeons	Learn Behav	37	4	289-98	Pigeon	na
Goldshmidt, J. N. and Fantino, E.	2004	Economic context and pigeons' risk-taking: an integrative approach	Behav Processes	65	2	133-54	Pigeon	na
Lagorio, C. H. and Hackenberg, T. D.	2012	Risky choice in pigeons: preference for amount variability using a token-reinforcement system	J Exp Anal Behav	98	2	139-54	Pigeon	Male
Lagorio, C. H. and Hackenberg, T. D.	2012	Risky choice in pigeons: preference for amount variability using a token-reinforcement system	J Exp Anal Behav	98	2	139-54	Pigeon	Male
Laude, J. R. and Beckmann, J. S. and Daniels, C. W. and Zentall, T. R.	2014	Impulsivity affects suboptimal gambling-like choice by pigeons	J Exp Psychol Anim Learn Cogn	40	1	44137	Pigeon	na
Laude, J. R. and Pattison, K. F. and Zentall, T. R.	2012	Hungry pigeons make suboptimal choices, less hungry pigeons do not	Psychon Bull Rev	19	5	884-91	Pigeon	na
Ludvig, E. A. and Madan, C. R. and Pisklak, J. M. and Spetch, M. L.	2014	Reward context determines risky choice in pigeons and humans	Biol Lett	10	8	1-5	Pigeon	na
McDevitt, M. A. and Diller, J. W. and Pietrzykowski, M. O.	2019	Human and pigeon suboptimal choice	Learn Behav	47	4	334-343	Pigeon	na
O'Daly, M. and Case, D. A. and Fantino, E.	2006	Influence of budget and reinforcement location on risk-sensitive preference	Behav Processes	73	2	125-35	Pigeon	na
Pattison, K. F. and Laude, J. R. and Zentall, T. R.	2013	Environmental enrichment affects suboptimal, risky, gambling-like choice by pigeons	Anim Cogn	16	3	429-34	Pigeon	na
Pisklak, J. M. and Madan, C. R. and Ludvig, E. A. and Spetch, M. L.	2019	The power of nothing: Risk preference in pigeons, but not people, is driven primarily by avoidance of zero outcomes	J Exp Psychol Anim Learn Cogn	45	4	431-445	Pigeon	na
Pisklak, J. M. and Madan, C. R. and Ludvig, E. A. and Spetch, M. L.	2019	The power of nothing: Risk preference in pigeons, but not people, is driven primarily by	J Exp Psychol Anim Learn Cogn	45	4	431-445	Pigeon	na

authors	year	title	journal	volume	issue	pages	Species	Sex
		avoidance of zero outcomes						
Shapiro, M. S. and Schuck-Paim, C. and Kacelnik, A.	2012	Risk sensitivity for amounts of and delay to rewards: adaptation for uncertainty or by-product of reward rate maximising?	Behav Processes	89	2	104-14	Starling	na
Smith, A. P. and Beckmann, J. S. and Zentall, T. R.	2017	Gambling-like behavior in pigeons: 'jackpot' signals promote maladaptive risky choice	Sci Rep	7	1	6625	Pigeon	na
Smith, Aaron P. and Zentall, Thomas R.	2016	Suboptimal choice in pigeons: Choice is primarily based on the value of the conditioned reinforcer rather than overall reinforcement rate	Journal of Experimental Psychology: Animal Learning and Cognition	42	2	212-220	Pigeon	na
Stagner, J. P. and Zentall, T. R.	2010	Suboptimal choice behavior by pigeons	Psychon Bull Rev	17	3	412-6	Pigeon	na
Zentall, T. R. and Andrews, D. M. and Case, J. P.	2017	Prior commitment: Its effect on suboptimal choice in a gambling-like task	Behav Processes	145		1-9	Pigeon	na
Zentall, T. R. and Andrews, D. M. and Case, J. P.	2017	Prior commitment: Its effect on suboptimal choice in a gambling-like task	Behav Processes	145		1-9	Pigeon	na
Zentall, T. R. and Stagner, J.	2011	Maladaptive choice behaviour by pigeons: an animal analogue and possible mechanism for gambling (sub-optimal human decision-making behaviour)	Proc Biol Sci	278		1203-8	Pigeon	na

4: Publications on gambling tasks in other species

authors	year	title	journal	volume	issue	pages	Species	Sex
Drezner-Levy, T. and Shafir, S.	2007	Parameters of variable reward distributions that affect risk sensitivity of honey bees	J Exp Biol	210		269-77	Honey Bee	na
Shafir, S. and Reich, T. and Tsur, E. and Erev, I. and Lotem, A.	2008	Perceptual accuracy and conflicting effects of certainty on risk-taking behaviour	Nature	453	7197	917-20	Honey bee	Female
van der Staay, F. and van Zutphen, Johanna A. and de Ridder, Mirjam M. and Nordquist, Rebecca E.	2017	Effects of environmental enrichment on decision-making behavior in pigs	Applied Animal Behaviour Science	194		14-23	Pig	Male
Murphy, E. and Kraak, L. and van den Broek, J. and Nordquist, R. E. and van der Staay, F. J.	2015	Decision-making under risk and ambiguity in low-birth-weight pigs	Anim Cogn	18	2	561-72	Pig	Male
